# Unassisted Switchable Dual‐Photoelectrode Devices Utilizing p‐n Carbon Quantum Dots as “Semiconductor Electrolytes”: Optimization Between H_2_O_2_ and Solar Electricity Production

**DOI:** 10.1002/advs.202417204

**Published:** 2025-05-19

**Authors:** Hui‐Min Duan, Chen‐Guang Li, Liu‐Meng Mo, Jing‐Shuang Dang, Xiao‐Hui Jia, Jia‐Cheng Yu, Yu‐Hang Mei, Anders Thapper, Hong‐Yan Wang

**Affiliations:** ^1^ Key Laboratory for Macromolecular Science of Shaanxi Province School of Chemistry and Chemical Engineering Shaanxi Normal University Xi'an 710119 China; ^2^ Department of Chemistry‐Ångström Laboratory Uppsala University P.O. Box 523 Uppsala 75120 Sweden; ^3^ Key Laboratory of Applied Surface and Colloid Chemistry Ministry of Education School of Chemistry and Chemical Engineering Shaanxi Normal University Xi'an 710119 China

**Keywords:** dual‐photoelectrode, electricity production, electrolyte optimization, H_2_O_2_ production, p‐n carbon dots

## Abstract

Switchable self‐driven photoelectrochemical (PEC) devices are developed to boost H_2_O_2_ or electricity generation under visible‐light illumination, in which p‐n type carbon quantum dots (N‐CQDs) is applied as conceptually‐new “semiconductor electrolytes”. The N‐CQDs contains N‐dopants, and both negatively‐ and positively‐charged surface groups. This allows N‐CQDs to act as the electrolyte and to interact with both a BiVO_4_ photoanode and a Cu_2_O photocathode. In a two‐compartment cell with a separating membrane, N‐CQDs can dynamically form p‐n heterojunctions with the photoanode or the photocathode, facilitating charge separation. In this setup, the fine‐tuned electronic structure of N‐CQDs promotes the two‐electron reactions with water or O_2_ to produce H_2_O_2_, achieving a rate of 28 µm min^−1^ and Faradic efficiency exceeding 80%. Switching into a one‐compartment cell, N‐CQDs promotes four‐electron charge transfer and stabilizes the photoelectrodes, giving electricity output for over 120 h. This control over electron transfer, selectivity, and durability cannot be achieved using traditional electrolytes.

## Graphical abstract

p‐n type carbon quantum dots (N‐CQDs) are designed as a conceptually‐new “semiconductor electrolyte” to develop a self‐driven photoelectrochemical cell that can be switched to optimize between H_2_O_2_ production and electricity generation under visible‐light illumination with water and O_2_ as reaction reagents. N‐CQDs can control electron migration, selectivity, and durability of the systems, which cannot be achieved using other traditional electrolytes.

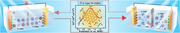

## 1 Introduction

The photoelectrochemical (PEC) technique aims to harvest incident light and perform energy conversation with zero‐carbon emissions at the source.^[^ ^1^ ^]^ As a green redox reagent and potential energy carrier, hydrogen peroxide (H_2_O_2_) has a wide range of applications in the chemical industry, environmental remediation, green synthesis, and fuel cell technology.^[^ ^2^ ^]^ The PEC process can efficiently convert the abundant and clean H_2_O or O_2_ into H_2_O_2_ through a water oxidation reaction (WOR) on a photoanode or a O_2_ reduction reaction (ORR) on a photocathode, giving a promising strategy for synthesizing H_2_O_2_.^[^ ^3^ ^]^ In addition, PEC devices can also be setup for electricity generation, and a PEC device equipped with dual‐photoelectrode has the potential to deliver more photovoltage, transforming both photon energy and chemical energy simultaneously into electricity.^[^ ^4^ ^]^ However, the PEC H_2_O_2_ production is usually accompanied by the decrease in the output of solar electricity in a dual‐photoelectrode system, because the formation of H_2_O_2_ involves a 2e^−^ transfer, which does not reach the maximum 4e^−^ transfer capability for solar electricity generation in neither ORR nor WOR. Therefore, we propose to develop a PEC device that can switch between modes to enhance either H_2_O_2_ production or electricity generation. However, a switchable PEC device presents significant challenges. First, from a thermodynamic standpoint, it is very difficult to sequentially extract electrons from water molecules due to the slow and energy‐demanding WOR.^[^ ^5^ ^]^ The cathodic ORR also has to overcome large overpotentials.^[^ ^6^ ^]^ Additionally, the 2e^−^ pathways for H_2_O_2_ generation is in direct competition with the 4e^−^ pathways, making it difficult to precisely control the selectivity of both the photoanode and photocathode.^[^ ^7^ ^]^ More importantly, boosting H_2_O_2_ generation or electricity output may involve different reaction mechanisms, making it challenging to simultaneously tune the two processes using the same semiconductor photoelectrodes and electrolytes. In addition, the compatibility of each side of the cell, as well as any cross talk between two sides also has to be considered. Therefore, there are only a few reports of dual‐photoelectrode PEC systems generating either electricity or H_2_O_2_, and to our knowledge, no dual‐photoelectrode PEC system has been reported that could optimize the generation of both simultaneously.^[^ ^8^ ^]^

A standard PEC cell consists of a semiconductor photoelectrode wired to a counter electrode, or dual tandem semiconductor photoelectrodes, both of which are submerged in an appropriate electrolyte solution.^[^ ^8^, ^9^ ^]^ The interaction between the electrolytes and photoelectrode surface gives an internal electric‐field across the semiconductor/electrolyte interface. Light absorption in the semiconductor materials generates charge carriers that can be regulated on the specific electrodes, and participate in different reactions. This highlights charge separation as the key step for the function of a PEC.^[^ ^3b^ ^]^ Fine‐tuning the electrolyte can affect the charge separation and charge injection efficiency into the electrodes. These electrolyte effects can sometimes even dominate the PEC activity and selectivity.^[^ ^10^ ^]^ However, electrolyte regulation is not well understood, and the main obstacle to tailor the PEC performance by electrolyte optimization is the lack of a universal protocol to achieve photo‐induced charge separation.^[^ ^11^ ^]^ Very recently, we pioneered the concept of “semiconductor electrolytes” by developing surface‐charged carbon quantum dots (CQDs) as both semiconductor and electrolyte in a PEC system.^[^ ^12^ ^]^ CQDs are low‐cost, light harvesting and 0 D carbon materials that can be dispersed in water as colloids owing to the surface‐anchored groups.^[^ ^13^ ^]^ We found that an aqueous solution containing only negatively surface‐charged CQDs was beneficial for PEC WOR with a n‐type semiconductor photoanode, while a solution with positively‐charged CQDs were matched to enhance PEC ORR on a p‐type photocathode.^[^ ^12^ ^]^ We demonstrated that the charged CQDs can act as electrolytes, directionally migrating in the solution when the PEC is operating.^[^ ^14^ ^]^ After approaching the photoelectrode surface, CQDs assembled into a heterojunction with the photoelectrode material.^[^ ^15^ ^]^ The appropriate band alignment facilitated the charge separation. In this sense, CQDs functioned as semiconductors.^[^ ^16^ ^]^ Accordingly, the suitable choice of “semiconductor electrolyte” for a particular photoelectrode can efficiently regulate the charge separation in the PEC process. Recent reports have also demonstrated the potential of CQDs to act as active sites for H_2_O_2_ generation based on WOR or ORR.^[^ ^17^ ^]^ Therefore, the design of CQDs that fit both the photoanodic and photocathodic side in a two‐compartment cell can facilitate the creation of a dual‐photoelectrode device to generate H_2_O_2_. When used in a one‐compartment cell, CQDs can improve the charge conductivity by their sp^2^ conjugated domain, which can direct the PEC process for electricity production.

Herein, we report a switchable self‐driven dual‐photoelectrode cell using the novel N‐doped CQDs (N‐CQDs) as “semiconductor electrolytes” for boosting H_2_O_2_ generation and electricity production. The N‐CQDs bears negative ─COO^−^ groups and positive ─N(CH_3_)_3_^+^ moieties on the surface, displaying either n‐ or p‐type conductivity.^[^ ^18^ ^]^ A PEC cell with a BiVO_4_ photoanode, a Cu_2_O photocathode, and the N‐CQDs in aqueous solution gives different outcomes depending on the cell configuration. In a proton exchange membrane‐separated two‐compartment cell, the N‐CQDs can integrate with each photoelectrode forming p‐n heterojunctions for charge separation.^[^ ^19^ ^]^ In this setup, the N‐CQDs mediates a 2e^−^ pathway for H_2_O_2_ production via PEC ORR and WOR under visible‐light irradiation. Switching to a one‐compartment cell without membrane, N‐CQDs promote 4e^−^ WOR on the photoanode and 4e^−^ ORR on the photocathode, giving an electricity output capable of powering a red LED for over 120 h without noticeable decay. This control over electron transfer pathways and selectivity regulation cannot be achieved using other normal electrolytes. Our work thus extends the use of conceptually new “semiconductor electrolyte” to a more practical dual‐photoelectrode cell, with clean, efficient, and low‐cost routes for sustainable solar electricity and valuable chemicals production.

## 2 Results and Discussion

### 2.1 Synthesis and Characterization of N‐CQDs

**Figure 1:**
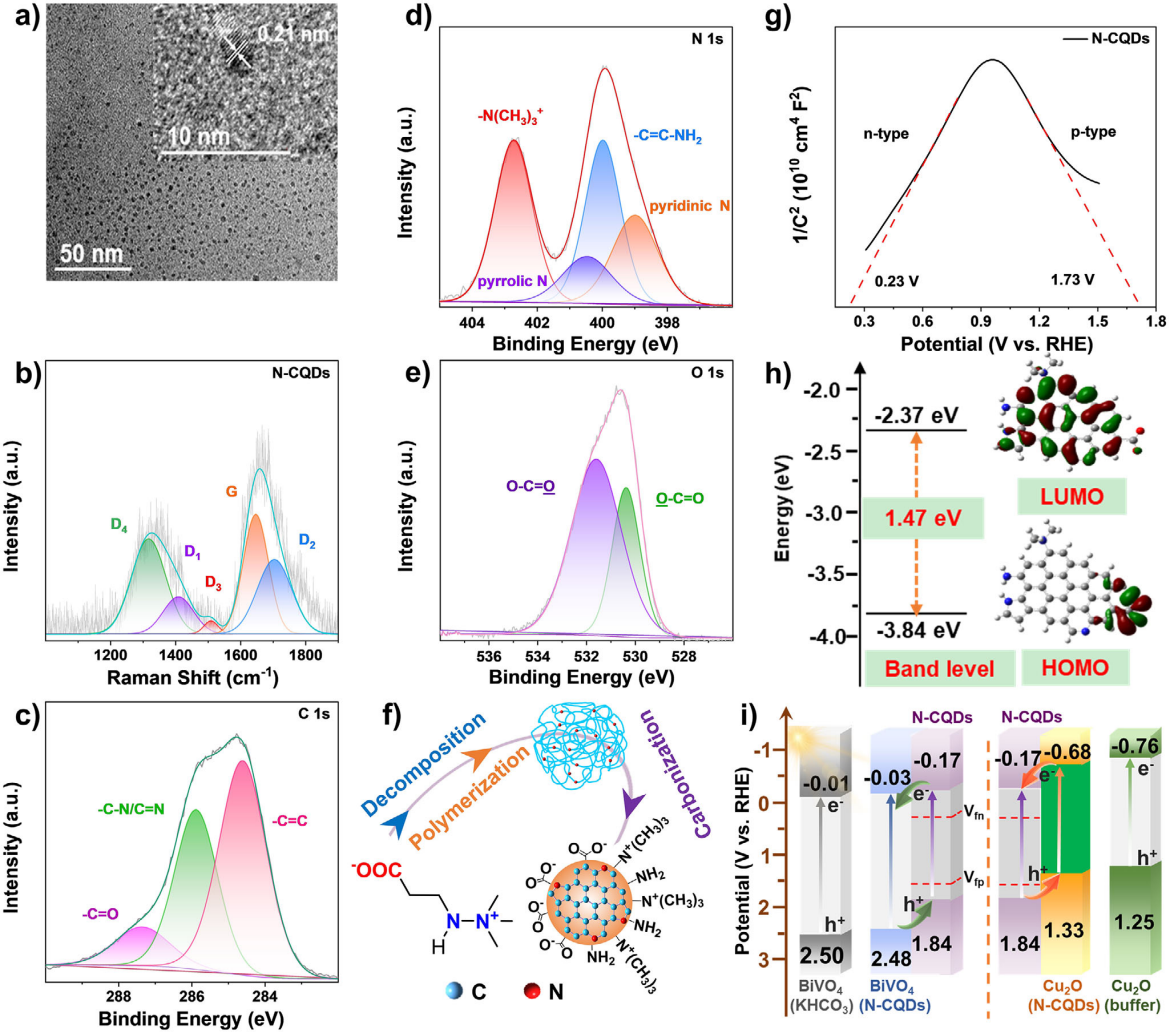
Characterization of the N‐CQDs. a) TEM spectra of N‐CQDs. b) Raman spectrum of N‐CQDs. High‐resolution c) C1s, d) N1s, and e) O1s XPS spectra of N‐CQDs. f) Schematic synthesis and proposed structure of N‐CQDs. g) Mott–Schottky plots of N‐CQDs showing the co‐existence of n‐ and p‐type conductivities. h) The band levels (left) and electron cloud distributions (right) of the LUMO and the HOMO from the DFT model of N‐CQDs. i) Energy band diagrams for p‐n junctions of N‐CQDs with BiVO_4_ and Cu_2_O respectively, and the band locations of BiVO_4_ in KHCO_3_ solution and Cu_2_O in citrate‐phosphate buffer solution. *V*_fn_ denotes the Fermi level corresponding to the n‐type semiconductor behavior of N‐CQDs, and *V*_fp_ represents the Fermi level associated with its p‐type semiconductor behavior. Both are taken from the intercept with the x‐axis in g).

The synthesis of N‐CQDs uses betaine‐type Meldonium as the precursor and aqueous ammonia as the nitrogen source in a hydrothermal‐assisted condensation polymerization at 180 °C for 5 h.^[^ ^20^ ^]^ The amount of reagents were determined based on the optimized H_2_O_2_ production, as described in the Supporting Information. Successive dialysis was used to isolate N‐CQDs as a brown yellow powder after freeze‐drying. However, the color quickly changes to dark brown and the N‐CQDs become sticky once exposed to ambient conditions, indicating the hydrophilicity of the material.^[^ ^21^ ^]^ High‐resolution transmission electron microscopy (HR‐TEM) shows that the N‐CQDs are uniform spherical nanoparticles with diameters of 3–6 nm, with a crystal spacing of 2.1 Å corresponding to the (100) plane in the graphitic regions (**Figure** **1**a).^[^ ^22^ ^]^ The X‐ray diffraction (XRD) spectrum (Figure S1, Supporting Information) shows a broad diffraction peak ≈23 °, correlating with a lattice spacing of 3.7 Å. This indicates an amorphous reassembled graphitic structure with short‐range order.^[^ ^23^ ^]^ The Raman spectrum (Figure 1b) shows a D band that can be deconvoluted into D1–D4 at 1410, 1705, 1510, and 1318 cm^−1^ respectively, accompanied by a G band centered at 1648 cm^−1^.^[^ ^24^ ^]^ The D2 and D4 bands correspond to the disordered graphitic lattice, and the G band represents the sp^2^ carbon structure with the ordered graphitic lattice.^[^ ^24^ ^]^ The D1 and D3 bands are related to carbon ring defects, generated from the carbon bonded to a heteroatom. This verifies that the nitrogen is successfully doped into the graphitic matrix in the N‐CQDs. Note that N‐doping can induce more catalytically active sites by the formation of electron‐rich or structural defects around the edges of CQDs. N‐doping is also favorable since it can adjust the semiconductor bandgap via tuning electronic properties with minimal structural alternations.^[^ ^18a^ ^]^ These two effects are expected to give positive impact on catalysis.^[^ ^25^ ^]^ X‐ray photoelectron spectroscopy (XPS) was used to provide insight into the surface functional groups and chemical components of the N‐CQDs. The high‐resolution C1s spectrum depicted in Figure 1c is resolved into three signals. The bonding energy at 284.6 eV belongs to C═C bonds.^[^ ^26^ ^]^ The accompanying peaks at 285.9 and 287.3 eV are assigned to C─N and C═O groups respectively.^[^ ^26^ ^]^ The N1s spectrum shows signals at 398.9, 399.9, and 400.4 eV with the respective atomic ratio of 22.2%, 31.8%, and 12.6% (Figure 1d), revealing the involvement of pyridinic N, C═C─NH_2_ and pyrrolic N in N‐CQDs.^[^ ^22^ ^]^ The appearance of a high percentage of pyridinic N and NH_2_ moieties implies that electrons are enriched in the N‐CQDs cores, which improves the charge conductivity.^[^ ^27^ ^]^ Interestingly, the clear signal at 402.7 eV in the N1s spectrum can be ascribed to a protonated quaternary ammonium species, showing the incorporation of N(CH_3_)_3_^+^ moieties on the N‐CQDs surface.^[^ ^28^ ^]^ The high‐resolution O1s spectrum (Figure 1e), indicates the existence of C═O and C─O bonds at 531.6 and 530.3 eV respectively, which confirms the presence of ─COO^−^ groups.^[^ ^29^ ^]^ In summary, positively charged ─N(CH_3_)_3_^+^ groups and negatively charged ─COO^−^ groups both decorate the N‐CQDs surface, and since the N‐CQDs have a Zeta potential of −12.3 mV (Figure S2, Supporting Information), the charges are almost completely neutralized. In Figure 1f, a model for the structure of N‐CQDs is proposed.

In order to characterize the semiconductor properties of the N‐CQDs, we conducted electrochemical impedance spectroscopic (EIS) measurements. Figure 1g shows the capacitance values of space charge region at various applied potentials for N‐CQDs. A Mott‐Schottky plot of 1/*C* ^2^ versus potential gives two straight lines with positive and negative slopes, in different potential regimes, which correlates with n‐ and p‐type conductivities, respectively (Figure 1g), confirming the co‐existence of both n‐ and p‐type conductivities in the N‐CQDs.^[^ ^18b^ ^]^ This indicates that the semiconductor type of CQDs can be flexibly regulated by the combination of surface‐modification groups and doped heteroatoms.^[^ ^18c^ ^]^ In order to investigate the synergistic effects of functional components on N‐CQDs electronic structure, we performed density functional theory (DFT) calculations by using a simplified model of the N‐CQDs based on a N‐doped sp^2^ hybrid carbon molecule with a ─COO^−^ and a ─N(CH_3_)_3_^+^ group. In addition, reference models, a N‐doped Csp^2^ molecule with a ─N(CH_3_)_3_^+^ group and a N‐doped Csp^2^ molecule with a ─COO^−^ group, were investigated respectively. As explained in the Supporting Information (Figure S3, Supporting Information), the calculations show a pair of orbital energy configurations, respectively designated as the highest occupied molecular orbital (HOMO) and lowest unoccupied molecular orbital (LUMO).^[^ ^18^, ^30^ ^]^ In Figure 1h, the left panel shows the band location from the DFT model of N‐CQDs, with the LUMO at −2.37 V and the HOMO at −3.84 V, giving a HOMO‐LUMO gap of 1.47 eV. The right panel shows the electron cloud distribution of the LUMO and the HOMO from the DFT model. As compared with the reference molecules in Figure S3 (Supporting Information), the LUMO orbitals are dominated by ─COO^−^ groups, related to the p‐type semiconductor feature. In contrast, the N‐doped Csp^2^ domains primarily contribute to the HOMO orbitals, indicating that the n‐type characteristics of N‐CQDs is mainly determined by electron transitions among the sp^2^ core and N‐dopant on the boundary.^[^ ^31^ ^]^ In this sense, N‐CQDs can provide a p‐n diode with the distributed p‐ and n‐type domains, which can make use of one of the two poles to interact with a photoanode or photocathode, respectively, and form a p‐n junction for charge separation.

We next evaluated the band levels of the N‐CQDs. Dispersing the N‐CQDs in water gives a transparent light‐brown solution, showing an absorption band from 200–300 nm assigned to conjugated π→π* (C═C) transitions, followed by a long trailing absorption into the visible light region (Figure S4, Supporting Information).^[^ ^32^ ^]^ Figure S5 (Supporting Information) exhibits the typical excitation‐wavelength dependence behavior for the luminescence from the N‐CQDs.^[^ ^33^ ^]^ Excitation of the N‐CQDs from 370–430 nm, gives a strong luminescence, implying that N‐CQDs can harvest the visible light for charge‐carrier generation. Based on the corresponding Tauc plots with (αh*v*)^2^ versus photon energy (h*v*), the band gap of N‐CQDs is determined as 2.01 eV (Figure S6, Supporting Information). Linear sweep voltammetry (LSV) in anodic and cathodic scan provides the HOMO and LUMO levels of N‐CQDs estimated as 1.81 and −0.17 eV (vs Reversible Hydrogen Electrode, RHE), respectively (Figure S7, Supporting Information),^[^ ^18c^ ^]^ giving an energy gap of 1.98 eV. This value agrees well with those obtained from optical absorption measurements. Extrapolating straight lines to the abscissa in the Mott–Schottky plot give two intercepts, representing the Fermi levels of the n‐type and p‐type semiconductor, located at 0.23 and 1.73 eV, respectively (Figure 1g).^[^ ^18b,c^ ^]^ Characterization of band levels for BiVO_4_ and Cu_2_O substrates in N‐CQDs are shown in Figures S8 and S9 (Supporting Information), respectively.^[^ ^34^, ^35^ ^]^ Combined with the p‐ and n‐type conductivity of N‐CQDs, it confirms the possible formation of type II p‐n junctions in both photoanodic (N‐CQDs and BiVO_4_) and photocathodic systems (N‐CQDs and Cu_2_O) (Figure 1i).^[^ ^36^ ^]^

### 2.2 PEC H_2_O_2_ Production with WOR on the Photoanode or ORR on the Photocathode in a Three‐Electrode Configuration Respectively

In order to monitor the WOR on the photoanode and ORR on the photocathode in PEC conditions, independent measurements was made in a three‐electrode configuration using the photoelectrode as a working electrode, Ag/AgCl as a reference electrode and a graphite rod as a counter electrode. The system was illuminated with commercial LEDs at λ = 450 nm with input optical power of 2.60 mW cm^−2^.

**Figure 2:**
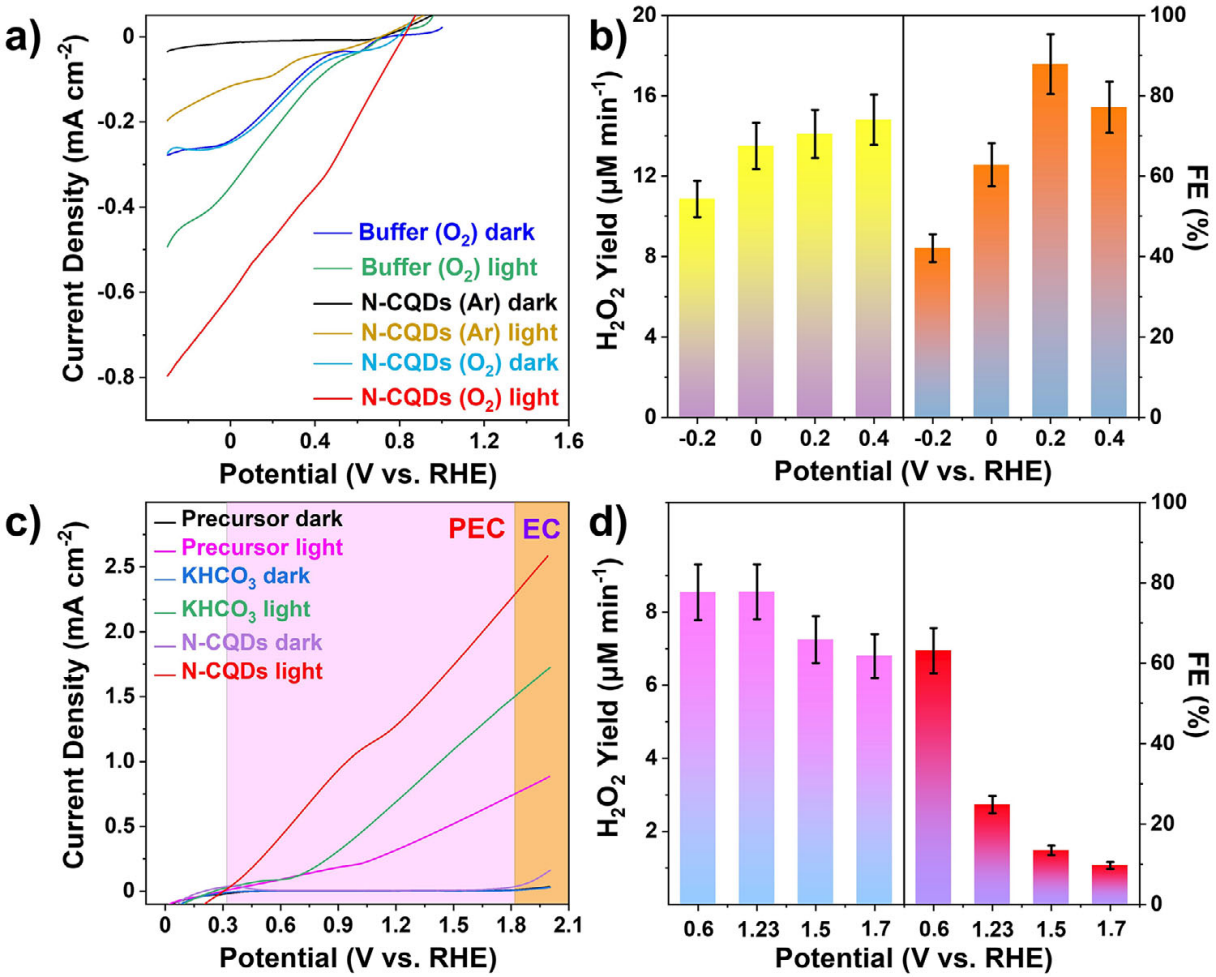
a,b) The ORR LSV and H_2_O_2_ generation of a Cu_2_O photocathode (2 cm^2^) in N‐CQDs or buffer solution in a three‐electrode configuration. c,d) The WOR LSV and H_2_O_2_ generation of a BiVO_4_ photoanode (2 cm^2^) in N‐CQDs, KHCO_3_ or precursor solution in a three‐electrode configuration. In (c) the PEC region starts at 0.32 V and the electrochemical (EC) region at 1.8 V given by the onset potential for N‐CQDs in light and dark, respectively.

In comparison, ORR is more complicated than WOR, and controlled by mass transfer and activation of O_2_. Therefore, only a few semiconductor materials have been shown to promote H_2_O_2_ production via PEC ORR until now.^[^ ^7^ ^]^ Cu_2_O is a typical p‐type semiconductor with an appropriate band gap to harvest visible light, making it a promising candidate for PEC reduction reactions.^[^ ^37^ ^]^ However, Cu_2_O usually suffers from serious light‐corrosion, but the stability can be dramatically improved by involving a p‐n junction.^[^ ^38^ ^]^ Therefore, the n‐type conductivity of N‐CQDs has potential to benefit for PEC ORR on Cu_2_O. Taken this into account, the performance of PEC ORR on Cu_2_O photocathode was investigated in an aqueous solution containing only N‐CQDs at almost neutral pH (pH 8). **Figure** **2**a shows the LSV curves on Cu_2_O in an Ar‐saturated N‐CQDs aqueous solution. In the dark, there is no response, but irradiation by LEDs gives a small photocurrent. In stark contrast, with the O_2_ saturated N‐CQDs solution applied, there is a strong photocurrent onset at 0.80 V, and the current keeps increasing with the potential, giving a saturated current of 0.82 mA cm^−2^ at −0.3 V. Therefore, the N‐CQDs can support a PEC ORR, which dramatically surpasses the response immersing Cu_2_O directly into an O_2_‐saturated commonly‐used citrate‐phosphate buffer or into a solution containing the precursors of N‐CQDs under identical pH and electro‐conductivity (Figure 2a; Figure S10 and Table S1, Supporting Information). Since H_2_O_2_ is susceptible to temperature, the PEC H_2_O_2_ generation over time is evaluated below 20 °C with continuous illumination at the selected bias for 10 min, by the titration method (Figure S11, Supporting Information).^[^ ^39^ ^]^ A Cu_2_O electrode can yield H_2_O_2_ with a rate of 10.9 ± 0.9 µm min^−1^ in an O_2_ saturated N‐CQDs solution at −0.2 V bias. Increasing the bias, the rate increases slightly reaching up to 14.8 ± 1.3 µm min^−1^ at 0.4 V, and a maximum Faradic efficiency (FE) of 87.9 ± 7.5% at 0.2 V (Figure 2b). While in buffer solution without N‐CQDs, only 0.34 ± 0.02 µm min^−1^ of H_2_O_2_ with a FE of 21.3 ± 1.3% is detected, showing the dramatic PEC enhancement when using the N‐CQDs solution (Figure S12, Supporting Information).

Furthermore, the independent measurement of PEC WOR was carried out on a BiVO_4_ photoanode.^[^ ^39^ ^]^ Directly immersing the BiVO_4_ photoanode in a N‐CQDs solution, shows a negligible current in the dark, along with a slight rise after 1.8 V, which is assigned to a electrochemical (EC) process for water oxidation. Upon LED illumination, a photocurrent onset at 0.32 V is observed, which increases with increasing potential, achieving a maximum of 2.27 mA cm^−2^ in the PEC region at 1.8 V (Figure 2c). The HCO_3_^−^ electrolyte is regarded as the most efficient media for 2e^−^ WOR for H_2_O_2_ production.^[^ ^40^ ^]^ However, the PEC response in the N‐CQDs solution outperforms that in the KHCO_3_ and the precursor solution of N‐CQDs, showing that the N‐CQDs are also excellent for supporting PEC WOR.^[^ ^12c^ ^]^ The maximum yield of H_2_O_2_ on the BiVO_4_ (2 cm^2^) electrode is determined as 8.5 ± 0.7 µm min^−1^ at 0.6 V in N‐CQDs solution, along with a FE of 70.1 ± 6.0%, but the FE gradually decreases with the potential, implying the decomposition of H_2_O_2_ on the anode with higher bias (Figure 2d; Figure S13, Supporting Information). In contrast, BiVO_4_ in a KHCO_3_ electrolyte delivers a H_2_O_2_ generation with a rate lower than 0.018 ± 0.001 µm min^−1^ under identical conditions. This may be due to the low efficiency of H_2_O_2_ generation in KHCO_3_ and consumption of H_2_O_2_ by HCO_3_^−^ electrolytes, as suggested recently in literature.^[^ ^8a^ ^]^

### 2.3 H_2_O_2_ Production from a Dual‐PEC Device in a Two‐Compartment Cell

**Figure 3:**
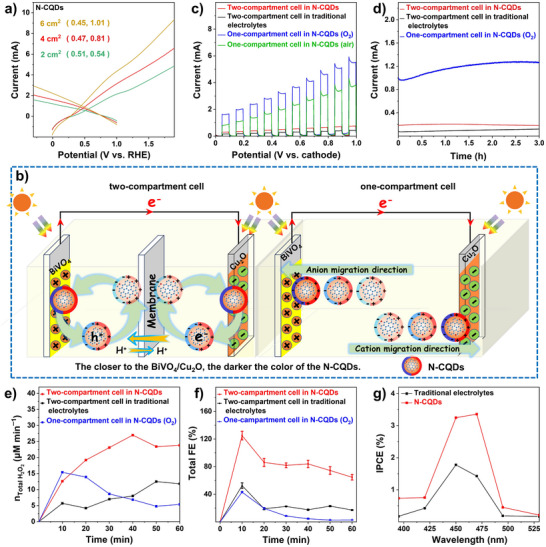
a) LSV intersections of photoanodic and photocathodic systems in a three‐electrode configuration. b) Schematic diagrams for the membrane‐separated two‐compartment cell and one‐compartment cell. c–g) LSV curves with chopped light irradiation, long‐time PEC operation, the sum of H_2_O_2_, FE generation, and IPCE on the photoanode and photocathode (6 cm^2^ electrode area) in different electrolytes and different cell setups.

LSVs of the photoanode and photocathode with the N‐CQDs electrolyte in a three‐electrode configuration interestingly shows that the individual current‐potential relationships display an intersection, giving an open‐circuit voltage (*V*_oc_) that shifts from 0.45 to 0.51 V with increasing electrode area (**Figure** **3**a). This indicates that the device can be self‐driven when illuminated.^[^ ^41^ ^]^ Therefore, we coupled a Cu_2_O photocathode and a BiVO_4_ photoanode in a self‐driven dual‐PEC device, enabling the electron‐triggered reduction and hole‐driven oxidation to proceed simultaneously, which utilizes the photons more efficiently. In order to maximize the incident light absorption and optimize the electrical power output, the Cu_2_O photocathode and BiVO_4_ photoanode with almost identical geometric areas are arranged into a parallel illumination configuration (Figure 3b).^[^ ^8b^ ^]^ Considering that a separating membrane may influence the output of the system, the device was studied in two different configurations: a two‐compartment electrolytic cell with a separating proton exchange membrane and a one‐compartment cell lacking the membrane (Figure 3b).

In a two‐compartment cell, the LSV curves with chopped light irradiation, suggests the current increasing proportionally to the potential under illumination, while staying close to 0 in the dark (Figure 3c). The periodic and stable photo‐response demonstrates the sensitivity of the system to light, and implies a flexible charge migration (Figure 3c).^[^ ^42^ ^]^ Extended photo‐electrolysis indicates stable photocurrents, displaying durability and stability for the PEC system (Figure 3d). Only small changes are observed in the UV–vis spectra of N‐CQDs electrolytes before and after PEC operation, excluding the decomposition of the N‐CQDs and detachment of semiconductor materials from the FTO plates (Figure S14, Supporting Information).

We next followed the self‐driven H_2_O_2_ generation in the two‐compartment cell. Using the N‐CQDs solution as the electrolyte can effectively promote ORR on Cu_2_O and WOR on BiVO_4_ electrodes, respectively. The sum of the rate of H_2_O_2_ production increases linearly with time for the first 40 min, reaching up to 28.0 ± 2.2 µm min^−1^ and a FE ≈80% (Figure 3e,f). In comparison, the yields of H_2_O_2_ with BiVO_4_ in KHCO_3_ solution and Cu_2_O in buffer solution are far lower with only 8.0 ± 0.4 µm min^−1^ at 40 min, associated with a FE ≈20%. This result agrees with the measurements with the independent three‐electrode systems. Incident photon‐to‐current conversion efficiency (IPCE) profiles were recorded under continuous illumination, and the N‐CQDs solution gives a higher value at each wavelength measurement (Figure 3g).^[^ ^43^ ^]^ However, switching into the one‐compartment cell, the H_2_O_2_ generation in N‐CQDs solution is sharply suppressed over the whole experiment, and is even lower than that of the two‐compartment cell in traditional electrolyte solutions (Figure 3e). In order to understand this behavior better, we turned our focus to the one‐compartment cell.

### 2.4 Electricity Generation in the Dual‐PEC Device in a One‐Compartment Cell

**Figure 4:**
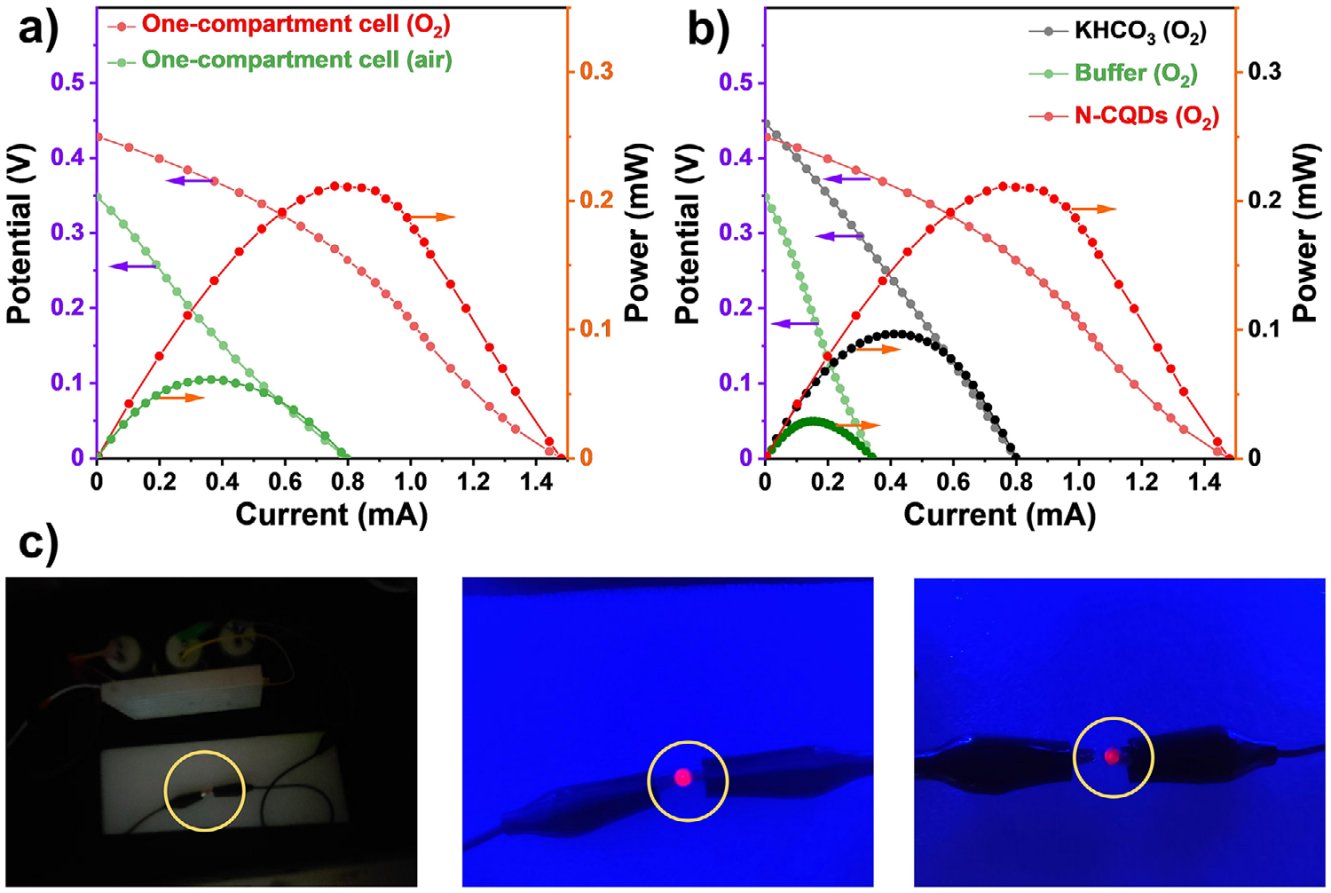
The *J–V* curve for the one‐compartment cell and calculated power density curve in a) N‐CQDs solution under air or O_2_‐saturated conditions, and b) in different O_2_‐saturated electrolyte solutions. c) The image for lightening a LED light by the light‐driven one‐compartment cell in N‐CQDs solution.

Interestingly, the one‐compartment cell with the N‐CQDs electrolyte can generate electricity, albeit with low H_2_O_2_ production. Under ambient conditions, the PEC cell with an electrode area of 6 cm^2^ gives a *P*_max_ of 0.06 mW, accompanied by an open‐circuit voltage (*V*_oc_) of 0.35 V and a short‐circuit current (*I*_sc_) of 0.79 mA. Purging O_2_ gas into the electrolyte solution, increases the *P*_max_ to 0.21 mW, associated with a *V*_oc_ of 0.43 V and a *I*_sc_ of 1.50 mA (**Figure** **4**a). The solar‐to‐electricity conversion efficiency *η*, is estimated as 0.11% after subtracting the dark power under O_2_‐saturated conditions (Table S2, Supporting Information). This result demonstrates that dissolved O_2_ is beneficial to the electrical power output. Using an O_2_ saturated KHCO_3_ or buffer electrolytes instead of N‐CQDs, gives a much lower electricity generation, with *P*_max_ of 0.09 and 0.03 mV, respectively (Figure 4b).

The electricity generation under light is further demonstrated by powering a commercial red LED light with a nominal voltage of 1.8–2.2 V. Three tandem PEC cells are connected in series as a proof‐of‐concept device. Under 450 nm light irradiation, the PEC device is able to power the red LED light (Movie S1, Supporting Information) with no perceptible decay in brightness over 120 h (Figure 4c), indicating the sustained solar electricity generation by the PEC device (Figure S15, Supporting Information). Natural sunlight could be also used for powering the LED light, but the brightness varies with the incident light intensity. When the PEC device is illuminated with a Xe lamp, the LED light is brighter (Figure S16, Supporting Information).

### 2.5 The Mechanism for the Two‐Compartment PEC Cell

**Figure 5:**
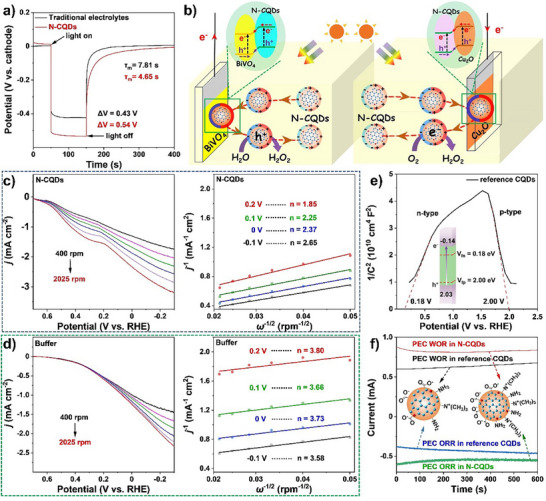
a) OCVD of the separated two‐compartment cell in different solutions. b) Mechanisms for the two‐compartment cell in N‐CQDs solutions. c,d) LSV curves and corresponding K‐L plots of the two‐compartment cell in N‐CQDs or KHCO_3_ buffer solutions respectively. e) Mott–Schottky plot and locations of the reference CQDs. f) The i‐t curves for photoanodic systems or photocathodic systems respectively in N‐CQDs solution or reference CQDs solution.

The above properties indicates that the N‐CQDs electrolyte mediates 2e^−^ migration for oxidizing water or reducing O_2_ on the respective photoelectrodes for H_2_O_2_ generation in the two‐compartment cell. When switching to a one‐compartment cell, electricity is generated as the dominant output. In order to clarify the reason behind these differences, the mechanism for each system was carefully studied. In the two‐compartment cell, the photoanodic side is composed of BiVO_4_ and a N‐CQDs solution, while the photocathodic side consists of Cu_2_O and a N‐CQDs solution. The schematic energy diagrams demonstrate that both sides can establish type II p‐n junctions by integrating the photoelectrode with the N‐CQDs solution, which is essential for charge separation (Figure 1i). Therefore, we extracted the photovoltage (ΔV) and the open circuit voltage decay (OCVD) from the photovoltage‐time (V‐t) spectrum for monitoring the charge separation in a two‐compartment cell.^[^ ^44^ ^]^ In the N‐CQDs solution, the cell presents a ΔV of 0.54 V, which decreases to 0.43 V when using traditional electrolyte systems (KHCO_3_ for the photoanode and citrate‐phosphate buffer for the photocathode) at identical conditions (**Figure** **5**a). The average charge decay lifetime of the V‐t profile provides a carrier lifetime *τ*_m_ of 4.65 s in the N‐CQDs solution, and 7.81 s in the traditional solutions.^[^ ^45^ ^]^ Significantly, the N‐CQDs solution gives rise to an accelerated charge carrier transfer, enhancing the PEC performance dramatically.

In order to monitor the possible transformation pathway in the two‐compartment cell, electron paramagnetic resonance (EPR) measurements were used to probe possible intermediates. On the photoanodic side, with 5,5‐dimethyl‐1‐pyrroline‐N‐oxide (DMPO) as a trapping agent, EPR signals are captured in the N‐CQDs solution under the PEC conditions after 10 min (Figure S17, Supporting Information). A sextet with an approximate intensity ratio of 1:1:1:1:1:1 (α_N_ = 15.4 G, α_H_ = 22.7 G) is identified and simulated with the Simfonia software, suggesting the formation of a DMPO‐C adduct.^[^ ^46^ ^]^ This is possibly derived from the N‐CQDs, which implies that N‐CQDs can attract the photo‐generated holes and then yield oxidized carbon radicals. Since the N‐CQDs keep their integrity after a long period of photoelectrolysis (Figure S14, Supporting Information), it implies that the oxidized N‐CQDs can return to their original state after water oxidation. A proposed mechanism is outlined in Figure 5b. As a typical n‐type semiconductor, the contact of BiVO_4_ with the electrolyte cause the energy band to bend upward. Light irradiation and excitation give the charge carriers. Photo‐generated electrons are repelled from the surface into the bulk of the BiVO_4_, liberating the holes accumulated on the photoanode surface, which results in a positively‐charged surface.^[^ ^47^ ^]^ This attracts the N‐CQDs particles via the negatively‐charged ─COO^−^ moieties. The N‐CQDs integrate with the BiVO_4_ into a type II p‐n heterojunction.^[^ ^12c^ ^]^ Along the energy alignment, holes migrates to N‐CQDs, accompanied by the formation of oxidized N‐CQDs, which are repulsed into the solution for water oxidation and then return to their original state. In this sense, the N‐CQDs colloids can be periodically adsorbed on or desorbed off the photoelectrode surface. An in‐situ UV–vis spectroelectrochemical investigation (Figures S18 and S19, Supporting Information) supports this proposal. The absorption of the N‐CQDs solution varies periodically during the experiment, demonstrating that the N‐CQDs can be dynamically transferred between the surface and the solution. This enables a sustainable supply of fresh N‐CQDs material, which increases the durability of the PEC system.^[^ ^12a,c^ ^]^ Additionally, note that no signal in the EPR measurements belongs to ·OH related species in Figure S17 (Supporting Information), suggesting that the location of the generated holes is higher than the potential for the H_2_O/·OH couple at 2.38 eV at pH 8, consistent with the proposal that the holes are moved from BiVO_4_ to the N‐CQDs along the aligned band levels.^[^ ^48^ ^]^

To investigate the superior activity of the N‐CQDs solution compared to buffer for PEC ORR, we carried out rotating disk electrode (RDE) measurements. Figure 5c,d illustrate the Koutecky–Levich (K─L) plot of Cu_2_O in N‐CQDs or buffer solution.^[^ ^49^ ^]^ The fitted lines are nearly parallel over the tested potential range, suggesting a first‐order reaction kinetics for ORR with respect to the dissolved O_2_ concentration. For each curve, almost similar electron transfer numbers (*n*) are obtained at different potentials. The value of *n* is calculated to be ≈2.0 in the N‐CQDs solution, but higher than 3.8 in buffer solution. This means that N‐CQDs solution is tuned to promote PEC ORR in a 2e^−^ pathway for H_2_O_2_ generation, but that a 4e^−^ process for water formation is dominant in buffer solution, in line with the higher selectivity for H_2_O_2_ formation in the N‐CQDs solution. Figure 5b shows the proposed mechanism for ORR in the N‐CQDs solution, in which the electrons migrate to the N‐CQDs across the Cu_2_O/N‐CQDs interface, leading to repulsion of the N‐CQDs into solution.^[^ ^12b^ ^]^ The reduced N‐CQDs activate the diffused O_2_ by their N‐doped sp^2^ clusters, which selectively promotes 2e^−^ transfer reducing O_2_ into H_2_O_2_,^[^ ^17b^ ^]^ followed by the recovery of N‐CQDs to their original state.

In the proposed mechanism, the N‐CQDs serves as the catalytic centers for both ORR and WOR. To support this proposal, reference CQDs were synthesized using the same procedure as that for N‐CQDs, except that the ammonia was absent, and the synthetical system was exposed to Ar. Although the reference CQDs can keep the p‐n feature and almost the same band edges with similar surrounding decorations on the surface as compared to N‐CQDs, the N‐dopants in the Csp^2^ cluster and boundary are dramatically decreased (Figure 5e; Figures S20 and S21, Supporting Information). As depicted in Figure 5f, the reference CQDs display more sluggish ORR and WOR activity under the same conditions as for the N‐CQDs. This means the structure changes strongly affect the PEC performance, implying the doped N‐CQDs may act as the real catalytic centers.^[^ ^50^ ^]^ Since the unique diode configuration of N‐CQDs, the energy level results into a favorable situation for electrons or holes injection into N‐CQDs, which leads to that electrons are located on the CB of BiVO_4_ on the photoanode and holes on the VB of Cu_2_O in the photocathodic side. In this sense, the electrons on BiVO_4_ are inclined to migrate through the external circuit and combine with holes on Cu_2_O for the output of electricity. As shown in Figure S22 (Supporting Information), the two‐compartment cell can output a *V*_oc_ at 0.28 V and a *I*_sc_ of 0.53 mA, along with the *P*_max_ of 0.037 mW, much lower than the one‐compartment cell. This may be coupled to the 2e^−^ charge migration capacity for H_2_O_2_ generation in the two‐compartment cell.

### 2.6 The Mechanism for the One‐Compartment PEC Cell

**Figure 6:**
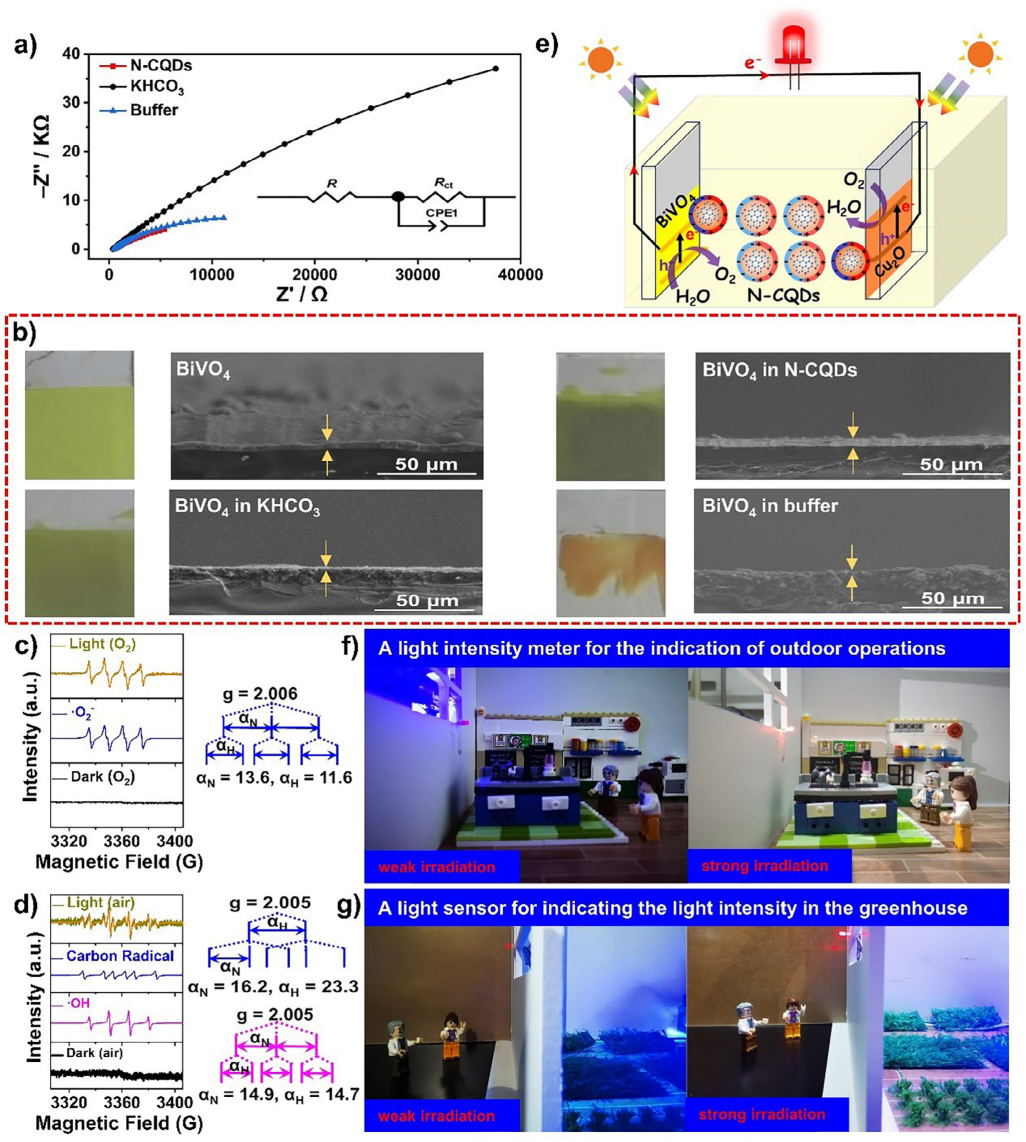
a) Fitted Nyquist plots of the EIS spectra for the one‐compartment cell in different solutions. b) SEM images of BiVO_4_ electrodes before (top left) and in different solutions after PEC operation (20 h) in a one‐compartment cell. c,d) EPR spectra of the BiVO_4_, N‐CQDs, and Cu_2_O modified FTO plate in DMSO or water respectively with DMPO as trapping agent. Note: The loose contact of components lead to the unavoidable formation of p‐n junctions between BiVO_4_ and N‐CQDs, therefore, holes were abstracted by N‐CQDs, and a minor signal ascribed to the DMPO‐C adduct was observed (α_N_ = 16.2 G, α_H_ = 23.3 G). (e) Mechanism of the one‐compartment cell in N‐CQDs solution. f,g) Potential application scenarios of the one‐compartment cell as a light indicator.

As shown in **Figure** **6**a, EIS demonstrates the cell in N‐CQDs solution provides the smallest arc diameter, correlated with the most efficient charge migration. The fitted Nyquist plots shows that the N‐CQDs electrolyte has the smallest charge transfer resistance, *R*_ct_ (Table S3, Supporting Information), demonstrating that the N‐doped sp^2^ domain in N‐CQDs can improve charge conductivity across the interface between the photoelectrodes and the electrolyte. Scanning electron microscope (SEM) and XPS show that the N‐CQDs can act as a bulk protector by electrostatic interaction with the electrodes to retard the light‐corrosion and swelling (Figure 6b; Figures S23 and S24, Supporting Information). Therefore, the N‐CQDs can give more stable and efficient electricity generation. In order to determine what catalytic event that takes place on the surface of the photoelectrodes, the possible intermediates were monitored by EPR. We prepared a well‐ordered system with BiVO_4_, N‐CQDs, and Cu_2_O layer‐by‐ layer modified on a FTO plate. The system was immersed in O_2_‐saturated DMSO and when illuminated, an EPR signal (α_N_ = 13.6 G, α_H_ = 11.6 G) is observed that is in agreement with the formation of O_2_^•‐^ (Figure 6c).^[^ ^51^ ^]^ In water, the system gives an EPR signal with a quartet with an approximate intensity ratio of 1: 2: 2: 1 (α_N_ = 14.9 G, α_H_ = 14.7 G), corresponding to a DMPO‐·OH adduct (Figure 6d), which demonstrates that water can be oxidized by the holes formed (E_H2O/·OH_ = 2.38 eV vs RHE, pH 8).^[^ ^48^ ^]^ This differed from the two‐compartment cell where no detectable signal assigned to ·OH species is detected in the photoanodic side (Figure S17, Supporting Information). Therefore, the holes are located on the more positive VB level of BiVO_4_ in the one‐compartment cell, positive enough to oxidize water into O_2_.

Figure 6e outlines a proposed mechanism for the one‐compartment cell. Upon illumination, the BiVO_4_ and Cu_2_O harvest photons and produce photo‐generated charges on the electrode surfaces, which can attract the surface‐charged N‐CQDs to directionally move between the BiVO_4_ photoanode and the Cu_2_O photocathode. The N‐CQDs act as the electrolyte and thanks to the excellent storage ability of the N‐CQDs, the charge migration is improved, as demonstrated by the EIS measurements (Figure 6a). The strong dependence on the O_2_ concentration for the electricity generation, and the formation of a DMPO‐·OH adduct observed with EPR spectroscopy, implies that a 4e^−^ pathway related to the H_2_O/O_2_ couple is involved in the PEC reaction. This suggests that the holes that accumulate on the BiVO_4_ electrode under irradiation are utilized at the solid/solution interface to oxidize water to O_2_. The O_2_ diffuse to the cathodic side, and is reduced by the surface‐located electrons on the p‐type Cu_2_O, transforming it into water. In a one‐compartment system, the absence of proton exchange membrane allows the N‐CQDs to freely move between the photocathode and photoanode. Moreover, the mass transfer of protons and O_2_ is also fast in the system, which leads to that the 4e^−^ pathway is the dominant process.^[^ ^52^ ^]^

In parallel, the electrons from the charge separation in BiVO_4_ migrate through the external circuit to combine with the liberated holes in the Cu_2_O photocathode to produce electricity. In this regard, the H_2_O/O_2_ couple act as a redox shuttle, similar to the I_3_^−^/I^−^ redox species in classic dye‐sensitized solar cells.^[^ ^53^ ^]^ In the one‐compartment configuration cell without membrane, the O_2_ molecules generated from WOR at the photoanodic side can diffuse rapidly to the cathodic region to enhance the concentration of dissolved O_2_ for improved ORR activity. Thus, self‐circulation of H_2_O‐O_2_‐H_2_O may afford a constant electricity power output over a long time. Since the O_2_ for the cathodic ORR can be obtained directly from air or from the product of WOR at the anodic side, the one‐compartment cell is capable to work in the open air. Because of the involvement of a 4e^−^ transfer process, the cell can deliver more electricity generation than the two‐compartment cell.

In this sense, the proton exchange membrane can adjust the selectivity of PEC system. However, if normal electrolytes are used, the 4e^−^ pathway should be dominant both in the one‐compartment form and the two‐compartment form (Figure 5d), because traditional electrolytes are unable to act as semiconductors. Therefore, the N‐CQDs electrolyte, together with the device configuration is important to modulate the PEC selectivity and activity. We also compared the performance of the device for H_2_O_2_ generation or electricity production with other reported unbiased dual‐photoelectrode systems (Table S4, Supporting Information). Clearly, our system can generate H_2_O_2_ and electricity at much milder conditions, and its performance is also above average compared with reported systems. More importantly, our system is unprecedented in using “semiconductor electrolytes” to efficiently tailor the selectivity for the PEC system output using different device setups. This highlights how rational design of electrolytes can contribute to sustainable development.

We also designed application scenarios for the switchable PEC cell. The two‐compartment cell with the N‐CQDs electrolyte can be applied to produce H_2_O_2_. Not only does the N‐CQDs show excellent selectivity in the PEC, but also the nanoparticle nature of N‐CQDs means that they can easily be isolated from the reaction system by centrifugation. Therefore, it would provide a convenient protocol to purify the H_2_O_2_. The related research is on‐going now. Additionally, the one‐compartment cell functions as a proof‐of‐concept PEC cell for electricity generation with an N‐CQDs aqueous electrolyte. The cell can tolerate moisture and outdoor conditions which can improve its applicability, e.g. as a light intensity meter (Figure 6f,g). Research into using CQDs as solid electrolytes, for a more powerful, durable, and portable PEC cell based on WOR and ORR, is on‐going. Together with developments on the photoelectrode side, this is expected to produce a system that is more economically feasible for large‐scale production.

## 3 Conclusion

In summary, we have successfully applied p‐n type N‐CQDs as a conceptually new “semiconductor electrolyte” to construct a self‐driven switchable PEC cell for either optimizing H_2_O_2_ or electricity generation under visible‐light illumination. The N‐CQDs are designed with N‐doping into Csp^2^ domains, bearing surface‐anchored negatively‐charged and positively‐charged groups. This allows the charged N‐CQDs to associate with both the BiVO_4_ photoanode and the Cu_2_O photocathode. In a separated two‐compartment cell with a proton exchange membrane, the N‐CQDs facilitates the unassisted 2e^−^ WOR and ORR for H_2_O_2_ generation at both the photoanode and photocathode. Switching to a one‐compartment setup without membrane, the cell can generate electricity under illumination, which powers a LED light for over 120 h without noticeable loss of performance. Mechanistic studies demonstrates that the p‐n diode configuration of N‐CQDs allows for the formation of type II heterojunctions with either the BiVO_4_ photoanode or the Cu_2_O photocathode in the two‐compartment cell, leading to efficient charge separation. The N‐CQDs act as the active sites and the N‐doped electronic structure assists in promoting the 2e^−^ pathway for H_2_O_2_ evolution from oxidizing water or reducing O_2_ at the photoanode and photocathode, respectively. In a one‐compartment setup without the membrane, the N‐CQDs can carry charge and improve the charge migration in the cell. This promotes a 4e^−^ transfer pathway, significantly increasing the electricity generation compared to a two‐compartment cell. The N‐CQDs also act as protectors for electrode surfaces from corrosion through electrostatic interactions, enabling a durable and stable system for electricity output. This work thus establishes a promising and feasible switchable PEC cell for the conversion of solar energy into chemicals and electricity based on electrolyte regulation.

## Conflict of Interest

The authors declare no conflict of interest.

## Supporting information



Supporting Information

Supplemental Movie 1

## Data Availability

The data that support the findings of this study are available from the corresponding author upon reasonable request.
