# Supplementary material for: Unassisted Switchable Dual‐Photoelectrode Devices Utilizing p‐n Carbon Quantum Dots as “Semiconductor Electrolytes”: Optimization Between H2O2 and Solar Electricity Production
Source: Adv Sci (Weinh). 2025 May 19;12(29):e17204. doi: 10.1002/advs.202417204 (PMC12362774; doi:10.1002/advs.202417204)
Supplement: Supplementary file 1 — Supporting Information [file ADVS-12-e17204-s001.docx]

Supporting Information

**Unassisted Switchable Dual-Photoelectrode Devices Utilizing p-n Carbon** **Quantum Dots as “Semiconductor Electrolytes”: Optimization Between** **H_2_O_2_ and Solar Electricity Production**

Hui-Min Duan^[a]^, Chen-Guang Li^[a]^, Liu-Meng Mo^[a]^, Jing-Shuang Dang^[c]^, Xiao-Hui Jia^[c]^, Jia-Cheng Yu^[a]^, Yu-Hang Mei^[a]^, Anders Thapper*^[b]^, Hong-Yan Wang*^[a]^

[a] Key Laboratory for Macromolecular Science of Shaanxi Province, School of Chemistry and Chemical Engineering, Shaanxi Normal University, Xi’an, 710119, China;

E-mail: [hongyan-wang@snnu.edu.cn](mailto:hongyan-wang@snnu.edu.cn)

[b] Department of Chemistry-Ångström Laboratory Uppsala University P.O. Box 523, 75120 Uppsala, Sweden;

E-mail: anders.thapper@kemi.uu.se

[c] Key Laboratory of Applied Surface and Colloid Chemistry, Ministry of Education, School of Chemistry and Chemical Engineering, Shaanxi Normal University, Xi’an 710119, China.

**Table of Contents**

1. Experimental details3
2. Table, Figures and Movie11
3. References46

**1. Experimental details**

**1.1 Materials**

The precursor for N-CQDs, betaine-type Meldonium, was purchased from Energy Chemical Co., Ltd. Bismuth nitrate pentahydrate, Vanadium oxide diacetyllacetone, hydrogen peroxide (30%) and acetic acid used for synthesis of BiVO_4_ were purchased from Sinopharm Chemical Reagent Co., Ltd. Peroxidase from horseradish and N,N-diethyl-p-phenylenediamine for the detection and quantification of H_2_O_2_ were purchased from Shanghai Aladdin Biochemical Technology Co., Ltd. All the reagents were used without any further purification. A cellulose dialysis membrane with molecular weight cut off (MWCO) of 500 Da was purchased from Sangon Biotech (Shanghai) Co., Ltd. FTO conductive glass plate was purchased from Luoyang Tengchang Xukun Biotechnology Co., Ltd.

**1.2 Preparation of photoelectrodes, N-CQDs and reference CQDs**

**1.2.1 Preparation of** **BiVO_4_ photoanode and Cu_2_O photocathode**

The BiVO_4_ covered FTO photoanode was prepared according to our published methods,^[^[^1^](#_ENREF_1)^]^and characterized by powder X-ray diffraction (XRD) shown in Figure S8a.

The preparation of the Cu_2_O covered FTO photocathode followed a modified protocol based on the literature,^[^[^2^](#_ENREF_2)^]^ and characterized by XRD shown in Figure S9a.

**1.2.2 Preparation of the BiVO_4_ photoanode and Cu_2_O photocathode**

All the carbon quantumdots (CQDs) samples were prepared based on a one-pot hydrothermal-assisted condensation polymerization method.^[^[^3^](#_ENREF_3)^]^

**N-CQDs**: N-CQDs were prepared with betaine-type Meldonium as the precursor and aqueous ammonia as the nitrogen source. The ratio and concentration of reagents were determined based on the optimized H_2_O_2_ production.^[^[^4^](#_ENREF_4)^]^ A concentration of the precursor is higher than 0.1 M, leads to the aggregation of CQDs, and no detectable H_2_O_2_ is produced when the CQDs are used in a PEC system. Lower concentration of precursor gives light yellow CQDs materials, but the production of H_2_O_2_ is very limited. The concentration of ammonia also has serious impact on the H_2_O_2_ generation. If more than 0.5 mL 0.1 M ammonia is added in the reaction system, the H_2_O_2_ yield from WOR sharply decreases. The H_2_O_2_ production for ORR displays a volcanic profile with the addition of 1 mL 0.1 M ammonia as the maximum. Typically, betaine-type Meldonium (0.3655 g, 2.5 mmol) was dissolved in 25 mL deionized water, with 0.5 mL 0.1 M ammonia solution added, which was kept in an autoclave at 180 °C for 5 h. The resulting brown system was filtered through a filter with pore diameter of 0.22 μm. Then, the solution was dialyzed by a cellulose dialysis membrane in 1 L deionized water under subsequent stirring. Every 4 h, fresh deionized water was applied and the whole dialysis process lasted for 2 days. The obtained solution was freeze-dried, which gaveN-CQDs as a light-yellow powder. The material is hydrophilic, andeasily becomes sticky with an associated color change to dark brown once exposed to room conditions.^[^[^5^](#_ENREF_5)^]^

**The reference CQDs**: The reference CQDs were synthesized using the same procedure as for the N-CQDs, except that the ammonia was deleted, and the synthesis was performed under Ar. The obtained CQDs were a light-yellow powder after purification and freeze-drying.

**1.3 Characterization**

High-resolution transmission electron microscopy (HR-TEM) images were collected on a Tecna iG2 F20 electron microscope at 200 kV. A Bruker D8 Advance X-ray powder diffractometer (XRD) was used to characterize the structures of materials. Raman spectra were measured on a Renishaw in Via with 532 nm laser source. X-ray photoelectron spectroscopy (XPS) analysis of the CQDs was carried out on a Kratos AXIS ULTRA DLD XPS instrument. The C 1s peak at 284.6 eV was chosen as the calibration for binding energy. UV-vis absorption spectra were recorded on a Hitachi U-3900 UV-vis Spectrophotometer. The diffuse reflection spectrum of the CQDs powder was also measured, and was converted from reflection to absorbance, on a Hitachi U-3900 UV-vis Spectrophotometer. The Photoluminescent (PL) spectra were recorded on a Hitachi F-7000 fluorescence spectrometer. Zeta potential measurements were conducted on a Malvern Nano ZS90 Particle Size Potentiometer. The pH of aqueous solutions was measured using an Orion star A211 pH meter from Thermo Fisher and electro-conductivity was measured on a DDSJ-319L conductivity meter.

**1.4 Electrochemical measurements**

Since strong electrolytes can complete dissociate in aqueous solution, it is easy to determine the charged particle concentration by controlling the concentration of electrolyte. However, the colloidal N-CQDs are weak electrolytes since the surface -COOH groups will not be completely deprotonated, which makes it difficult to identify the accurate concentration. Based on the Kohlausch's law of independent ion movement, the concentration of charged particles is proportional to the electro-conductibility of the system.^[^[^6^](#_ENREF_6)^]^ Therefore, in order to compare the activity of a N-CQDs solution with traditional electrolytes (KHCO_3_ and citrate-phosphate buffer) under similar conditions, the conductivity and pH was kept almost identical in both the systems (Table S1).

**1.4.1 Photoelectrochemical (PEC) measurements**

The electrochemical experiments were operated on a CH instruments electrochemical analyzer (model CHI660E) at ambient conditions, unless otherwise stated. The system was irradiated using a 450 nm LED lamp with the input optical power of 2.60 mW cm^−2^. Before the PEC O_2_ reduction reaction (ORR), O_2_ was continuously pumped into the aqueous solution with the respective electrolyte present, for 30 minutes. The photocurrent density was determined by dividing photocurrent by photoelectrodes areas.

**Three-electrode system**: In order to monitor the PEC capability of WOR on the photoanodic system and ORR on the photocathodic system for producing H_2_O_2_, an independent measurement was initiated in a three-electrode configuration with the photoelectrode as a working electrode, Ag/AgCl as a reference electrode and graphite rod as a counter electrode. The photoelectrode was prepared using a 5 cm^2^ FTO electrode covered with 2 cm^2^ semiconductor material. The N-CQDs solution can be applied as electrolyte for both WOR and ORR, while KHCO_3_ aqueous solution was used for WOR and citrate-phosphate buffer solution was used for ORR as references. The potentials reported in the work are converted to Reversible Hydrogen Electrode (RHE) scale via calibration using the following equation: *E* (vs RHE) = *E* (vs Ag/AgCl) + 0.197 + 0.0591 × pH. Unless otherwise stated, all potentials are reported versus RHE. The pH and electro-conductivity were adjusted to be almost identical in the electrolyte solutions used, as shown in Table S1.

**Table S1.** The pH and conductivity of N-CQDs, KHCO_3_, citrate-phosphate buffer, and reference CQDs solution.

|  | N-CQDs | KHCO_3_ | Citrate-Phosphate Buffer | reference  CQDs |
| --- | --- | --- | --- | --- |
| pH | 8.8 | 8.6 | 8.6 | 8.6 |
| Conductivity (mS·cm^-1^) | 2.7 | 2.6 | 2.6 | 2.7 |

**Two-electrode system**: The two-electrode system was equipped with a BiVO_4_ photoanode and a Cu_2_O photocathode (each photoelectrode was prepared using a 10 cm^2^ FTO electrode covered with 6 cm^2^ semiconductor material). In the two-compartment cell with a proton exchange membrane, the N-CQDs solution can be used as electrolyte for both the photoanodic side and the photocathodic side. As reference, a KHCO_3_ solution was used as electrolyte for the photoanodic side and a citrate-phosphate buffer solution was used for the photocathodic side. In the one-compartment cell without membrane, N-CQDs, KHCO_3_, and buffer solution were used for the comparison. The pH and electro-conductivity were adjusted to be almost identical in the electrolyte solutions used, as shown in Table S1.

**1.4.2 Solar-to-electricity energy conversion**

The solar-to-electricity energy conversion efficiency (*η*) is calculated according to equation (S1-2):^[^[^7^](#_ENREF_7)^]^

$\text{η}\text{ = }\frac{\text{P}_{\text{out}}}{\text{P}_{\text{in}}}\text{= }\frac{\text{V}_{\text{oc}}\text{ × }\text{I}_{\text{sc}}\text{ × }\text{FF}/{\text{A}_{\text{illuminated}}}}{\text{P}_{\text{hν}}}$ (S1)

$\text{FF}\text{ = }\text{P}_{\text{max}}\text{·}\text{I}_{\text{sc}}^{\text{ -1}}\text{·}\text{A}_{\text{illuminated}}\text{· }\text{V}_{\text{oc}}^{\text{ -1}}$ (S2)

Here, *P*_out_ is the electrical power output. *P*_in_ represents the photochemical energy input, and *P*_hν_ is the incoming photon energy, respectively. *A*_illuminated_ means the area of the electrodes exposed to the illumination, in this work this was the sum of *A*_BiVO4_ and *A*_Cu2O_. *V*_oc_ is the open-circuit voltage, *I*_sc_ is the short-circuit current, and *FF* is the fill factor. Note that *η* was calculated by using the maximum power density with the dark curve set as zero current. The solar-to-electricity conversion efficiency of the two-compartment cell and the one-compartment cell is shown in Table S2.

**Table S2**. The solar-to-electricity conversion efficiency of the two-compartment cell and the one-compartment cell.

|  | *V*_oc_ (V) | *I*_sc_  (mA) | *P*_max_  (mW) | *ŋ*  (%) |
| --- | --- | --- | --- | --- |
| Two-compartment cell  (with the photoanodic system in air; the photocathodic system in O_2_) | 0.28 | 0.53 | 0.037 | 0.02 |
| One-compartment cell (air) | 0.35 | 0.79 | 0.06 | 0.03 |
| One-compartment cell (O_2_) | 0.43 | 1.50 | 0.21 | 0.11 |

**1.4.3 Rotating disk electrode (RDE) measurements**

RDE measurements were performed in an O_2_-saturated N-CQDs solution or citrate-phosphate buffer solution. Linear sweep voltammetry (LSV) was operated with a scan rate of 10 mV s^−1^, accompanied by the electrolysis on a rotating ring electrode at 0.9 V vs. Ag/AgCl. The LSV curves were collected with different rotation rates of 400 rpm, 625 rpm, 900 rpm, 1225 rpm, 1600 rpm and 2025 rpm, respectively. The electron transfer numbers (*n*) can be calculated at different potentials; -0.1 V, 0 V, 0.1 V and 0.2 V, respectively, based on Koutecky-Levich (K-L) equation S3-4:^[^[^8^](#_ENREF_8)^]^

$\frac{\text{1}}{\text{j}}\text{= }\frac{\text{1}}{\text{j}_{\text{k}}}\text{+ }\frac{\text{1}}{\text{K}}\text{· }\frac{\text{1}}{\text{ω}^{\text{1}/\text{2}}}$ (S3)

$\text{K}\text{=0.620}\text{n}\text{F}\text{A}\text{C}_{\text{0}}\text{D}^{\text{2}/\text{3}}\text{ν}^{\text{-1}/\text{6}}$ (S4)

*j*: the current density measured in the experiment, mA·cm^-2^

*j*_k_: the kinetically controlled current density of RDE, mA·cm^-2^

*ω*: rotating speed of rotation disk electrode, rpm

*n*: the number of transferred electrons in ORR

F: faraday constant, 96485 C·mol^-1^

*A*: area of electrode, cm^2^

*C*_0_: the concentration of oxygen in solution, pH = 5~13 [0.0012 mol·cm^-3^]

*D*: diffusion rate of oxygen molecules, pH = 5~13 [1.9×10^-5^ cm^2^·s]

*ν*: dynamic viscosity of solution, 0.01 cm^2^·s

**1.4.4** **The measurement of Mott-Schottky (M-S) plots**

The purified N-CQDs or reference CQDs were dissolved in water, giving a concentrated N-CQDs or CQDs aqueous solution, respectively, which was further drop-casted onto a FTO plate and then air-dried at room temperature. The modified FTO plates were then dried in a vacuum oven at 100 ^o^C for 1 h. M-S measurements were conducted in a three-electrode system, in which the working electrode was the N-CQDs/FTO plate, the counter electrode was a graphite rod, the reference electrode was the Ag/AgCl, and 0.1 M KHCO_3_ was the electrolyte. The CHI 660E electrochemical work station was used for M-S tests. The flat-band potentials (*V*_fn_ and *V*_fp_) of p-n type N-CQDs were determined using M-S plots at potentials varying from 0.3 V to 1.5 V (vs. RHE) with a frequency of 1 kHz, while the reference CQDs were measured from 0.2 to 2.2 V. Based on the M-S equation shown in S5, a plot of $\text{1}/{\text{C}_{\text{SC}}^{\text{2}}}$ against *E* yields a straight line. The values of *V*_fn_ and *V*_fp_ were determined from the intercept on the E axis.

$\frac{\text{1}}{\text{C}_{\text{SC}}^{\text{2}}}\text{ = }\text{}\frac{\text{2}}{\text{ε}\text{ε}_{\text{0}}\text{E}\text{N}_{\text{d}}}\text{(}\text{E}\text{-}\text{E}_{\text{fb}}\text{-}\frac{\text{k}_{\text{B}}\text{T}}{\text{e}}\text{)}$ (S5)

Here, *C*_SC_ is the interfacial capacitance, *ε* is the dielectric constant of the semiconductor, *ε*_0_ is the permittivity of free space, *N*_d_ is the number of donors, *E* is the applied voltage, *E*_fb_ is the flat-band potential of semiconductor, *e* is the electronic charge, *k*_B_ is Boltzmann’s constant and T is the absolute temperature. The flat-band potential can be approximated as the Fermi level (*V*_f_) in the semiconductor.^[^[^9^](#_ENREF_9)^]^ A positive slope indicates an n-type semiconductor, while a negative slope indicates a p-type semiconductor.

**1.4.5** **The calculation of the** **average charge decay lifetime in the Open circuit voltage decay (OCVD) test**

The calculation of the average charge decay lifetime was based on a reported method in the literature.^[^[^10^](#_ENREF_10)^]^ The OCVD test was conducted to characterize the effect of N-CQDs solution on the charge transfer efficiency, which gives a curve of photogenerated potential as a function of time. After light-off, the curve suddenly decays and reach its value before the irradiation. A second-order nonlinear fitting is performed on the decay curve, and the average charge decay lifetime (*τ*_m_) of the system was calculated using the following formula (S6).

$\text{τ}_{\text{m}}\text{= }\frac{\text{τ}_{\text{1}}\text{ × }\text{τ}_{\text{2}}}{\text{τ}_{\text{1}}\text{ + }\text{τ}_{\text{2}}}$ (S6)

Here, *τ*_1_ and *τ*_2_ is the fitting lifetime based on the second-order nonlinear fitting respectively.

**1.4.6** **The in-situ spectroelectrochemical measurement**

The in-situ UV-vis spectroelectrochemical measurements were performed to follow the absorption of N-CQDs solution at a specific wavelength during the PEC. ‌The three-electrode PEC system with the photoelectrode as a working electrode, Ag/AgCl as a reference electrode, graphite rod as a counter electrode and N-CQDs solution as electrolyte, was mounted in a quartz cuvette. The concentration, the pH and conductivity of N-CQDs solution was identical to the normal PEC measurement. ‌Under illumination with a 450 nm LED light, a constant-potential chronoamperometry was conducted under constant potential of 1.23 V (vs. RHE) for the WOR and 0.4 V (vs. RHE) for the ORR. Every few minutes, the absorption of the N-CQDs solution was recorded at the specific wavelength.

**1.5 The determination and quantification of H_2_O_2_ in the PEC system**

A titration method was applied to determine and quantify the production of H_2_O_2_,^[^[^11^](#_ENREF_12)^]^ using the mixture of N,N-diethyl-1,4-phenylene-diamine (DPD) and peroxidase (POD), which were prepared separately. DPD (0.1 g) was dissolved in 10 mL 0.05 M H_2_SO_4_ solution. POD (10 mg) was dissolved into 10 mL deionized water, which was kept in the refrigerator for storage. Before PEC operation, the UV-vis spectrum for the mixture of 1.0 mL electrolysis solution, 0.025 mL potassium phosphate buffer solution (1 M), 1.5 mL water, 0.025 mL DPD solution and 0.025 mL POD solution was collected. And then, 1.0 mL electrolysis solution was collected after the PEC process had been running for 10 min, which was further added with 0.2 mL potassium phosphate buffer solution (1 M), 1.5 mL water, 0.025 mL DPD solution and 0.025 mL POD solution. The system was well mixed and analyzed by UV-vis spectroscopy. The detection of H_2_O_2_ was based on the differences in UV-vis spectroscopy before and after the PEC process. The quantification of H_2_O_2_ was carried out using a job plot for the mixture of 1.0 mL H_2_O_2_ solution with different concentrations, 0.2 mL potassium phosphate buffer solution (1 M), 1.5 mL water, 0.025 mL DPD and 0.025 mL POD solution.

**1.6 Density functional theory (DFT) calculations**

All geometry optimization was carried out based on spin polarized DFT calculations using Gaussian G16 software. PBE0 functional and SMD continuum solvation model was applied with water as the solvent. For the geometry optimization, all elements utilized the 6-31+g (d) basis sets. Moreover, the molecular orbital was post-processed and visualized by Gaussview.

**2. Tables and Figures**

Figure S1. XRD spectrum of N-CQDs.

Figure S2. Zeta potential measurement of the N-CQDs (0.1 M) at 25 ^o^C.

**a)**


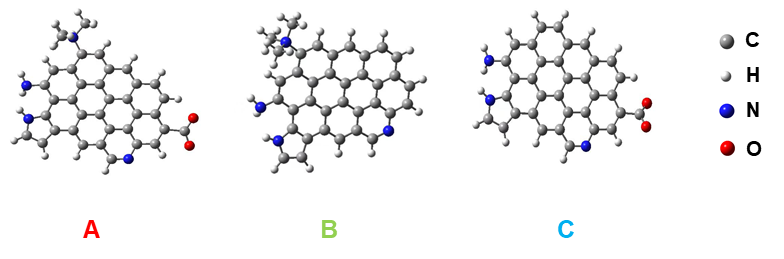


**b)**


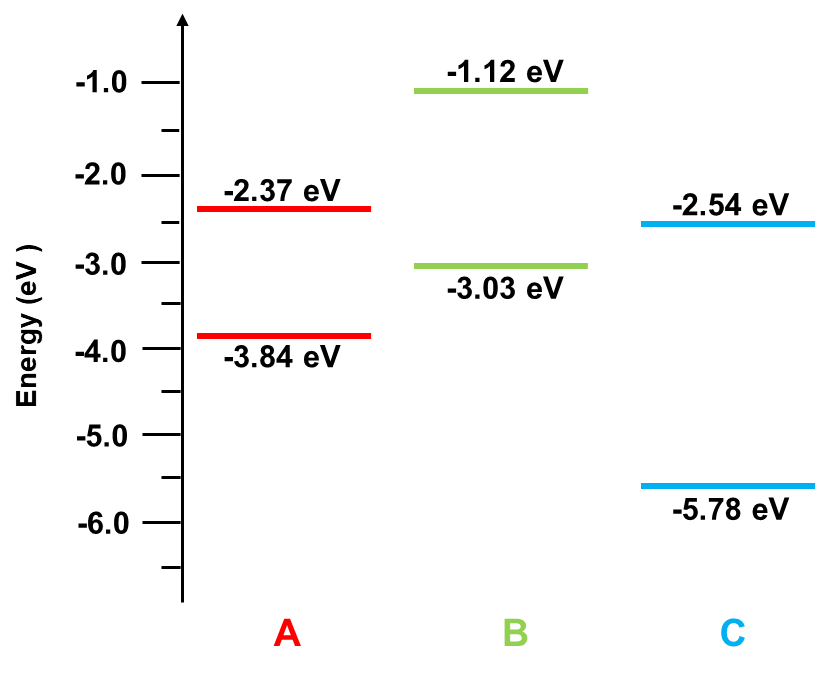


**Figure S3.** DFT calculations to investigate the effects of functional groupson the N-CQDs electronic structure. a) The structure of model (A) and reference (B-C) molecules. b) the highest occupied molecular orbital (HOMO) and lowest unoccupied molecular orbital (LUMO) of A-C based on the DFT calculations.^[^[^12^](#_ENREF_13)^]^

In order to investigate the effects of the functional groups on the N-CQDs semiconductor behavior, we conducted density functional theory (DFT) calculations of a N-doped sp^2^ hybridized carbon molecule with -COO^-^ and -N(CH_3_)_3_^+^ groups as a simplified model (A). In addition, DFT calculations were made on two reference molecules, a N-doped Csp^2^ molecule with only a-N(CH_3_)_3_^+^ group (B) and a N-doped Csp^2^ molecule with only a -COO^-^ group (C).

Compared with B, the HOMO level of A is negatively shifted by 0.81 eV, and the LUMO by 1.25 eV. Therefore, the change for the LUMO is more significant after the incorporation of -COO^-^ groups in the model. Since the LUMO correlates with the ability to obtain electrons, it contributes to p-type conductivity, which means that electrons can be accumulated from the sp^2^ conjugated domain in the molecule to the carboxylic acid functional groups. Comparing A with C, the change for the HOMO level is more significant, which implies that the incorporation of -N(CH_3_)_3_^+^ can seriously impact the oxidation ability of the molecule. In this sense, electrons are transferred from the -N(CH_3_)_3_^+^ functional group to the sp^2^ conjugated layer, contributing to the n-type conductivity.^[^[^13^](#_ENREF_14)^]^

**Figure S4.** UV-vis spectrum of an aqueous solution of N-CQDs.

**Figure S5.** Luminescence spectra at different wavelengths of an aqueous solution of N-CQDs.

**Figure S6.** a) UV-vis DRS spectrum and b) the corresponding Tauc plot of N-CQDs.

**Figure S7.** The LSV spectra of N-CQDs in the cathodic scan a) and anodic scan b) respectively, which was carried out in the corresponding N-CQDs solution with bare FTO electrode as working electrode, Ag/AgCl electrode as reference and graphite rod as a counter electrode.

The LSV curves afford the HOMO and LUMO of N-CQDs at 1.81 eV and -0.17 eV respectively, giving an energy gap of 1.98 eV. This value agrees well with that obtained from optical absorption. In order to determine the band levels for both N-CQDs and photoelectrodes, the HOMO or LUMO level of N-CQDs is based on the potentials from the electrochemical measurements, and the other band location can be calculated in the combination of *E*_g_ obtained from optical absorption. Here, since the LUMO is located at -0.17 V for N-CQDs, it leads to HOMO at 1.84 eV based on 2.01 eV of energy band read from theUV-vis DRS spectrumin Figure S6.

**Figure S8.** a) The XRD spectrum of BiVO_4_. b) The corresponding Tauc plot of BiVO_4_ based on UV-vis DRS spectrum. c-d) Mott-Schottky plots of BiVO_4_ photoanode in N-CQDs c) or KHCO_3_ d) aqueous solution at different frequencies.

Based on the UV-vis DRS spectrum, the corresponding Tauc plot of BiVO_4_ affords an Energy gap (*E*_g_) of 2.51 eV. In the N-CQDs solution, BiVO_4_ exhibits positive slopes in Mott-Schottky plots, matching the feature for n-type semiconductor. The flat band potential (*V*_fb_) of BiVO_4_ can be determined as the intercept on the abscissa, with the value of -0.03 eV. Since a n-type semiconductor has a negligible gap between *V*_fb_ and bottom edge of Conduction Band (CB),^[^[^14^](#_ENREF_16)^]^ the CB of BiOV_4_ in N-CQDs solution is estimated as -0.03 eV, which accordingly gives the Valence Band (VB) level as 2.48 eV. Similarly, the CB of BiVO_4_ in KHCO_3_ solution is determined as -0.01 eV, which accordingly gives the VB level located at 2.50 eV.

**Figure S9.** a) The XRD spectrum of Cu_2_O. b) The corresponding Tauc plot of Cu_2_O based on UV-vis DRS spectrum. c-d) Mott-Schottky plots of Cu_2_O photoanode in N-CQDs aqueous solution c) or citrate-phosphate buffer solution d) at different frequencies.

Based on the UV-vis DRS spectrum, the corresponding Tauc plot of Cu_2_O provides an *E*_g_ of 2.01 eV. In the N-CQDs solution, Cu_2_O shows a negative slope in a Mott-Schottky plot, consistent with the p-type semiconductor feature. Moreover, the intercept on the abscissa gives *V*_fb_ of Cu_2_O at 1.33 eV. Since p-type semiconductors have a negligible gap between *V*_fb_ and the upper edge of the VB, the VB of Cu_2_O in the N-CQDs solution is estimated as 1.33 eV, and the CB level as -0.68 eV.^[^[^15^](#_ENREF_17)^]^ Similarly, the CB of Cu_2_O in buffer solution is determined as 1.25 eV, which affords the CB level as -0.76 eV.

**Figure S10.** The ORR LSV of a Cu_2_O photocathode in N-CQDs or precursor (betaine-type meldonium) aqueous solution in a three-electrode configuration.

**Figure S11.** a) The UV-vis absorption spectra for the mixture with 1 mL H_2_O_2_ solution with different concentration (0.01 M, 0.02 M, 0.04 M, 0.06 M, 0.08 M, 0.1 M), 0.2 mL potassium phosphate buffer solution (1 M), 1.5 mL water, 0.025 mL DPD and 0.025 mL POD solution. b) The job plot for the quantification of H_2_O_2_ based on the absorption in the spectra at 551 nm.

**Figure S12.** UV-vis absorption spectra of a mixture with a) 1.0 mL O_2_-saturated N-CQDs solution or b) citrate-phosphate buffer, 0.2 mL potassium phosphate buffer solution (1 M), 1.5 mL water, 0.025 mL DPD solution and 0.025 mL POD solution before and after PEC ORR on the Cu_2_O photocathode in a three-electrode configuration at different applied bias for 10 min.

**Figure S13.** UV-vis absorption spectra of a mixture with a) 1.0 mL N-CQDs or b) KHCO_3_ solution, 0.2 mL potassium phosphate buffer solution (1 M), 1.5 mL water, 0.025 mL DPD solution and 0.025 mL POD solution before and after PEC WOR on the BiVO_4_ photocathode in a three-electrode configuration at different applied bias for 10 min.

**Figure S14.** a) UV-vis absorption spectra of N-CQDs electrolyte solution before and after PEC ORR in photoanodic system and PEC WOR in photocathodic system for 3 h in a two-compartment system. b) UV-vis absorption spectra of N-CQDs electrolyte solution before and after PEC operation for 3 h in a one-compartment system.


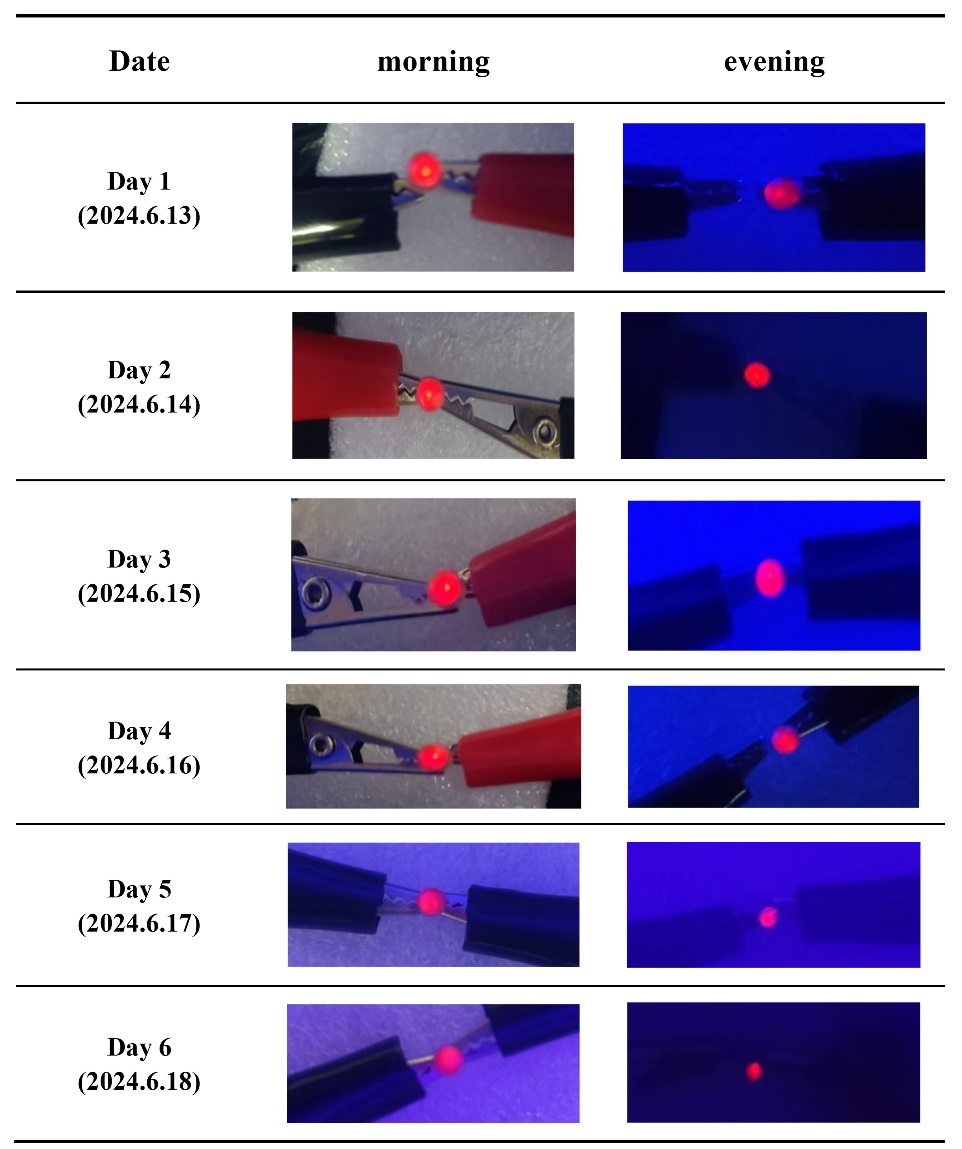


**Figure S15.** The images of the device for lighting a red LED light under the sustainable illumination of commercial LEDs (λ at 450 nm with input optical power of 2.60 mW cm^−2^) for over 120 h.


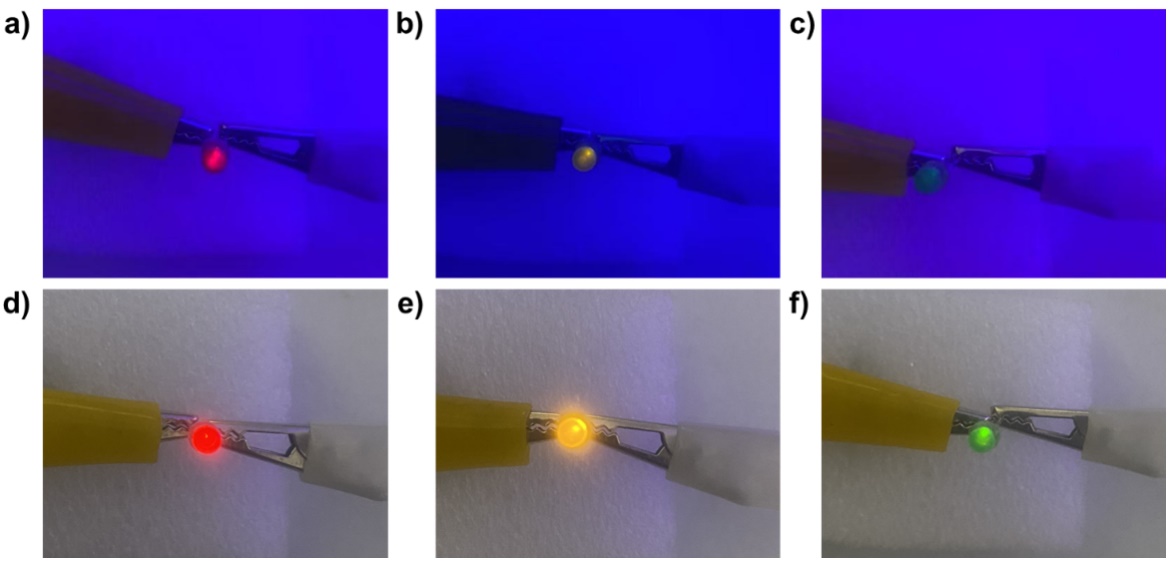


**Figure S16.** a-c) Images of a red, yellow, and green LED light, respectively, lightened by a one-compartment cell in N-CQDs solution irradiated by a LED light (λ = 450 nm, input optical power of 2.60 mW cm^−2^); (d-f) Images of a red, yellow, and green LED light, respectively, lightened by a one-compartment cell in N-CQDs solution irradiated by a Xe lamp (input optical power of 871.00 mW cm^−2^ with a cutoff wavelength of 435 nm).

In Figure S16a-c, the LED light (λ = 450 nm, input optical power of 2.60 mW cm^−2^) was used as the irradiation source to illuminate the BiVO_4_ photoanode and Cu_2_O photocathode in a N-CQDs solution, which can generate electricity to lighten up an external LED light. Figure S16a shows an external red LED light, S16b is a yellow LED light, and S16c is a green LED light. It is clear that the green LED light requires for more power to be lit up. In comparison, a Xe lamp (input optical power of 871.00 mW cm^−2^) with a cutoff wavelength of 435 nm was then used as the irradiation source. It can also light up the external LED lights, and all of them are much brighter than when the 450 nm LED light was the irradiation source (Figure S16d-f). Therefore, it shows that the Xe lamp with much higher input optical power gives much brighter external LED lights.


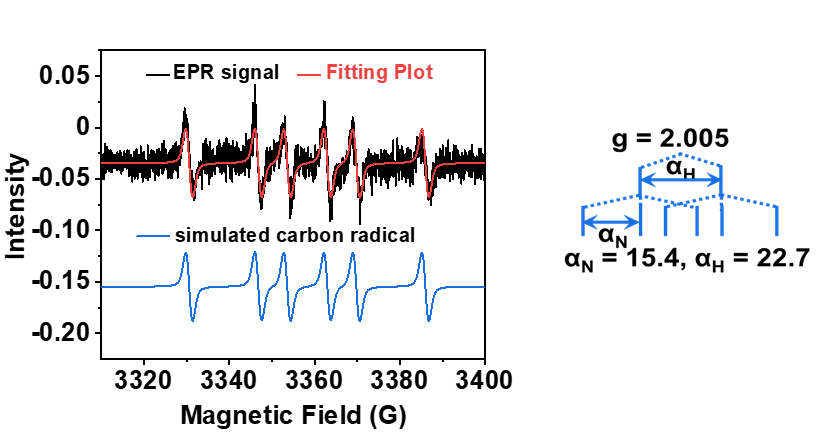


**Figure S17.** Experimental EPR spectrum (black) and simulated spectrum (blue) of the photoanodic system in the two-compartment cell with BiVO_4_ substrate in N-CQDs solution, and DMPO (2 μM) after PEC WOR operation for 10 min.

**Figure S18.** The in-situ UV-vis spectroelectrochemical absorption at 400 nm for the photoanodic system in N-CQDs solution.

**Figure S19.** The in-situ UV-vis spectroelectrochemical absorption at 340 nm for the photocathodic system in N-CQDs solution.

**Figure S20.** a) The corresponding Tauc plot of reference CQDs based on UV-vis DRS spectrum. b) The LSV spectra of reference CQDs in the cathodic scan, which was carried out in the reference CQDs solution with bare FTO electrode as working electrode, Ag/AgCl electrode as reference and graphite rod as a counter electrode.

It is clear that the reference CQDs bears the similar p-n type conductivity as N-CQDs based on the Mott-Schottky measurement in Figure 5e.^[^[^16^](#_ENREF_18)^]^ Extrapolating straight lines in the Mott-Schottky plot to the abscissa gives two intercepts, representing Fermi levels of n-type or p-type semiconductor locate at 0.18 eV and 2.00 eV respectively. Based on the corresponding Tauc plots with (αh*v*)^2^ versus photo energy (h*v*), the band gap of the reference CQDs is evaluated as 2.17 eV in Figure S20a.^[^[^9^](#_ENREF_9)^]^ Applying LSV in cathodic scan, it gives the LUMO level of the reference CQDs estimated at -0.14 eV in Figure S20b. Therefore, the HOMO of reference CQDs is located at 2.03 eV. The band location of reference CQDs is shown in Figure 5e.^[^[^16^](#_ENREF_18)^]^

**
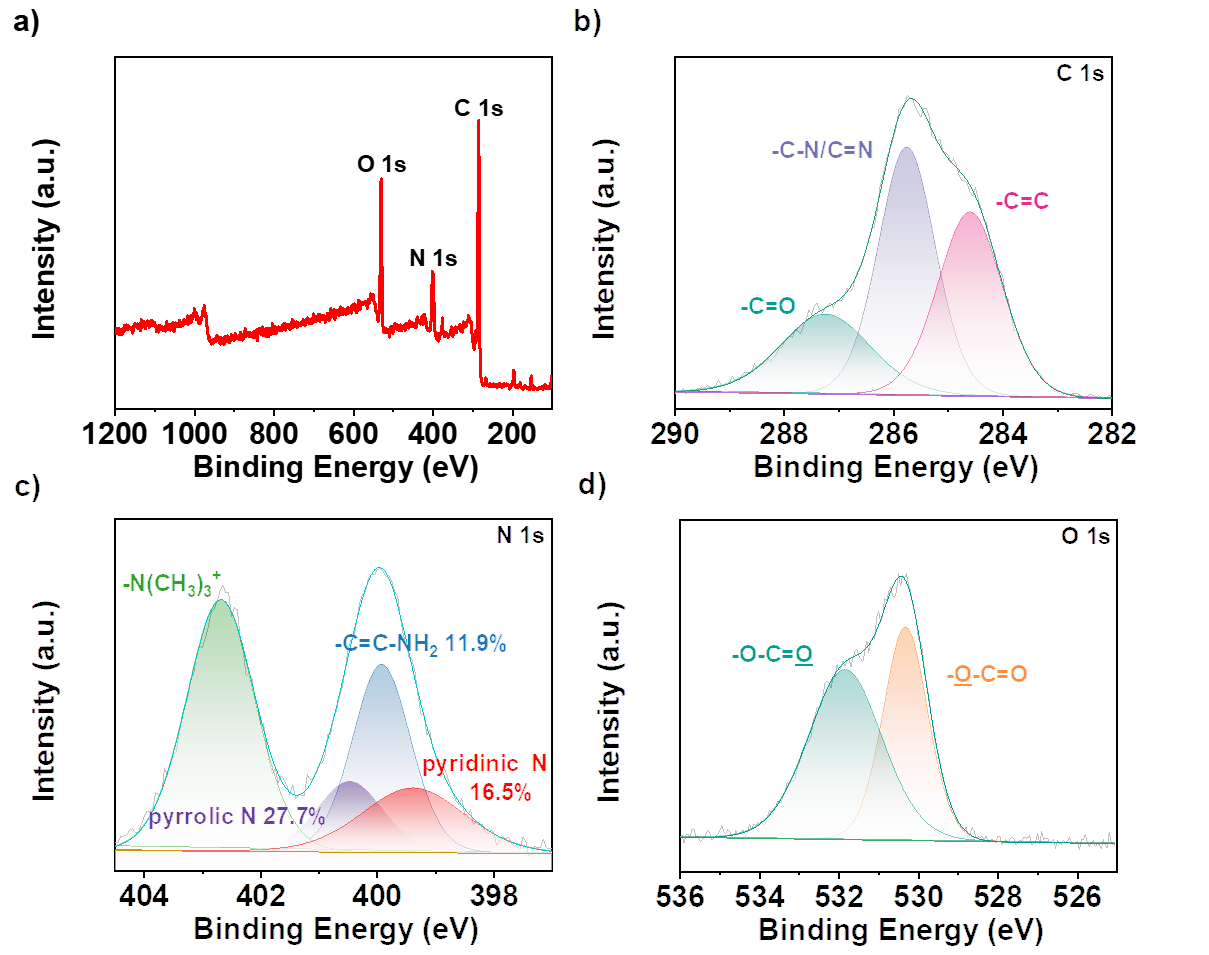
**

**
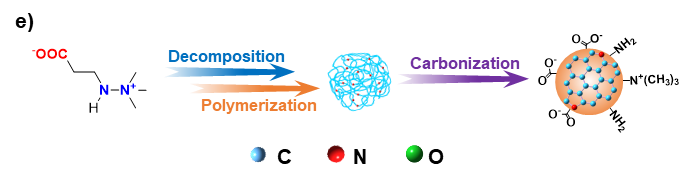
**

**Figure S21.** a-d) XPS survey, high-resolution C 1s, N1s, O1s spectra of the reference CQDs, respectively. e) The proposed reference CQDs structure.

The full-range XPS spectrum in Figure S21a shows a high atomic content of O, C and N respectively, implying -COO^-^ and -N(CH_3_)_3_^+^ groups from the Meldonium precursor may remain and cover the reference CQDs surface. The high-resolution C1s spectra is shown in Figure S21b, and could be resolved in three signals. The band at 284.6 eV belongs to C=C bonds, evidencing the Csp^2^ conjugated environment.^[^[^17^](#_ENREF_19)^]^ The accompanying peaks at 285.9 eV and 287.3 eV are assigned to C-N or C=N groups, respectively.^[^[^18^](#_ENREF_20)^]^ The N1s spectrum in Figure S21c shows signals at 398.9 eV, 399.9 eV and 400.4 eV with the respective atomic ratio of 16.5%, 27.7% and 11.9%, revealing the involvement of pyridinic N, C=C-NH_2_ and pyrrolic N in reference CQDs.^[^[^19^](#_ENREF_21)^]^ While in N-CQDs, the proportions of them are 22.2% and 31.8% and 12.6% respectively. Clearly, the N-CQDs possesses more electron-rich or structural defects on the surface, which can act as the active sites for both WOR and ORR. Interestingly, a clear signal at 402.7 eV ascribed to a protonated quaternary ammonium species is also observed in the N1s spectrum, which suggests that N(CH_3_)_3_^+^ moieties are incorporated in the reference CQDs surface.^[^[^20^](#_ENREF_22)^]^ The O1s high-resolution spectrum (Figure S21d), which indicates C=O and C-O bond at 531.6 and 530.3 eV, respectively, confirms the presence of -COOH groups.^[^[^21^](#_ENREF_23)^]^ Consequently, the proposed reference CQDs structure is shown in Figure S21e.

**Figure S22**. The *J*-*V* curve and the calculated power density curve for the two-compartment cell under O_2_-saturated condition.

**
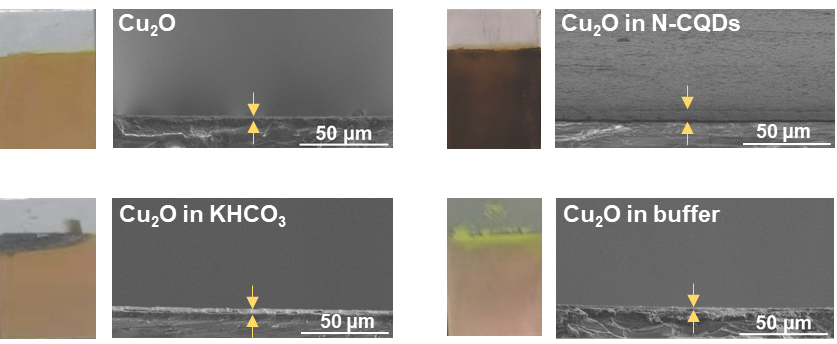
**

**Figure S23**. SEM images of Cu_2_O electrodes in different solutions before and after 20 h PEC operation in a one-compartment cell.

**
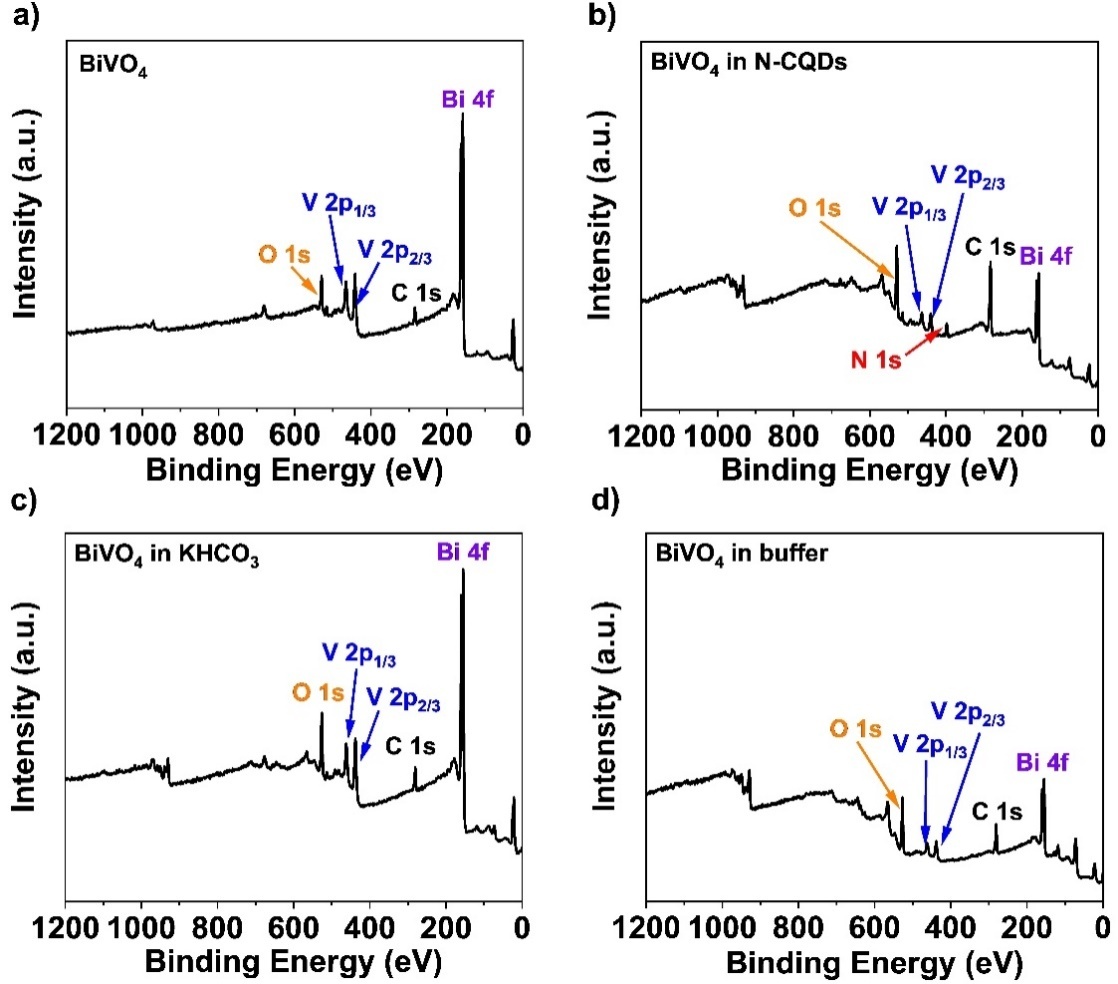
**

**
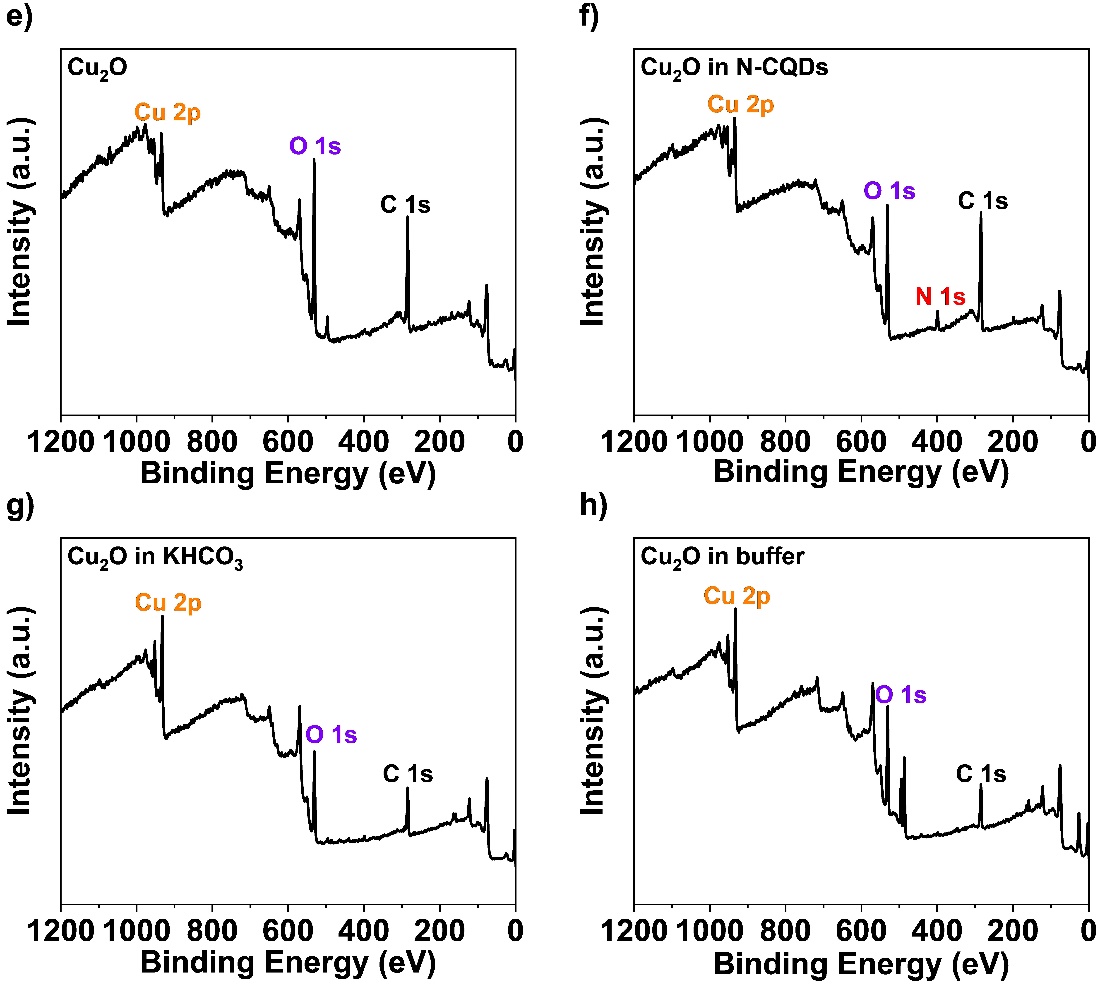
**

**
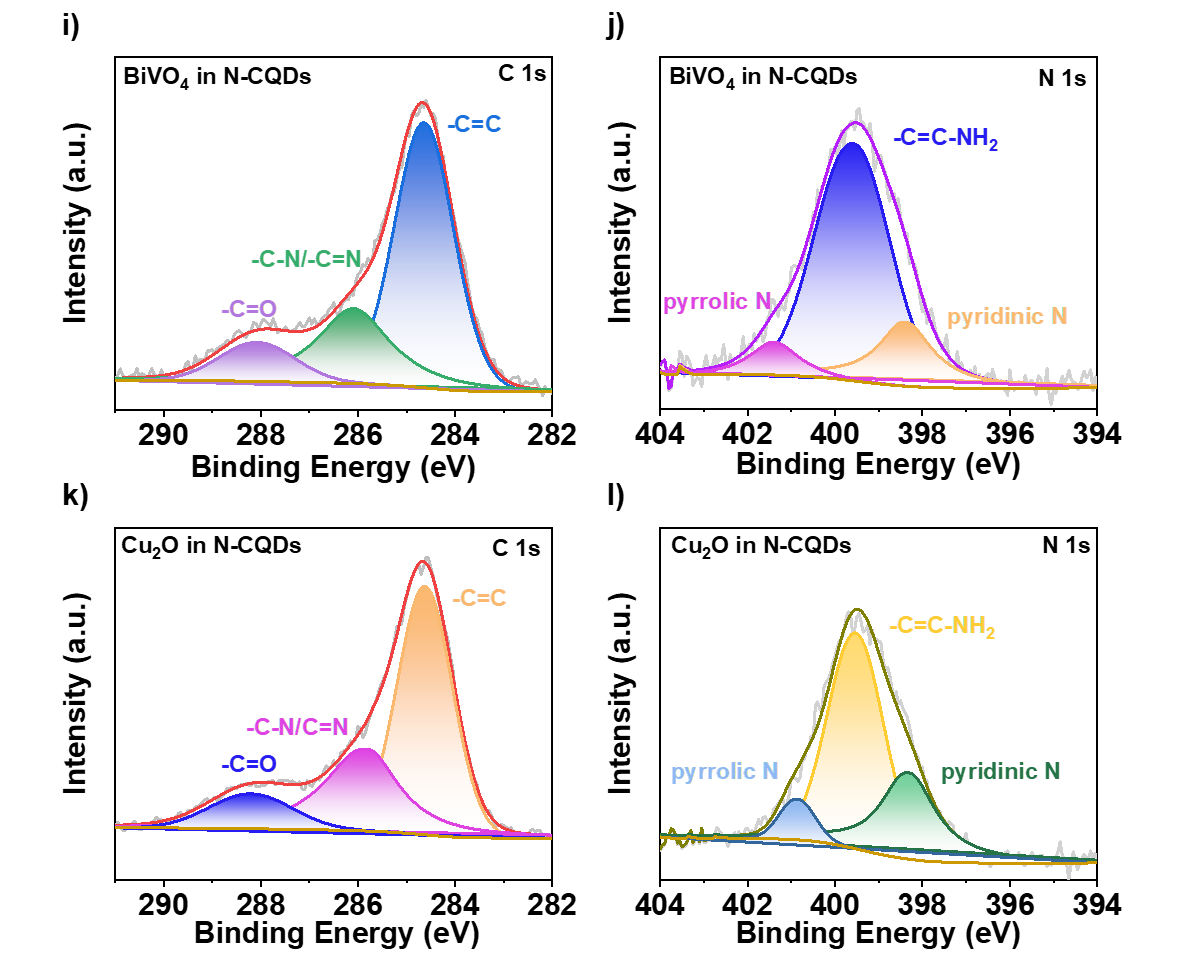
**

**Figure S24**. The XPS spectra of photoelectrode surface after PEC operation in one-compartment cell with different solutions as electrolytes. a-h) The full scan of XPS spectra for BiOV_4_ and Cu_2_O in different electrolyte solutions. i-j) The high-resolution C1s, N1s XPS spectra for BiOV_4_ in N-CQDs. k-l) The high-resolution C1s, N1s XPS spectra for Cu_2_O in N-CQDs.

The full scan of BiVO_4_ and Cu_2_O in different electrolyte solutions are shown in Figure S24a-h. It is clear that only the electrodes in N-CQDs shows clear N1s signals and enhanced C1s peaks, implying N-CQDs particles can cover both the BiVO_4_ and the Cu_2_O surface. The high-resolution C1s spectra of BiVO_4_ in N-CQDs solution is depicted in Figure S24i, can be resolved into three signals. The band at 284.6 eV belongs to C=C bonds, evidencing a Csp^2^ conjugated environment.^[^[^17^](#_ENREF_19)^]^ The accompanying peaks at 286.1 eV and 288.1 eV are assigned to C-N or C=O groups, respectively.^[^[^18^](#_ENREF_20)^]^ The N1s spectrum of BiVO_4_ in the N-CQDs solution shows signals at 398.4 eV, 399.6 eV and 401.4 eV, revealing the presence of pyridinic N, C=C-NH_2_ and pyrrolic N, respectively (Figure S24j).^[^[^19^](#_ENREF_21)^]^ These signals are consistent with those in the C1s and N1s spectra of N-CQDs (Figure 1c and 1d). Figure S22k shows the high-resolution C1s spectra of Cu_2_O in N-CQDs solution. The peaks at 284.6 eV, 285.9 eV and 288.2 eV are assigned to C=C, C-N or C=O groups respectively.^[^[^17-18^](#_ENREF_19)^]^ Interestingly, some obvious signals at 398.3 eV, 399.5 eV and 400.9 eV ascribed to pyridinic N, C=C-NH_2_ and pyrrolic N are also observed in N1s spectrum of Cu_2_O in N-CQDs solution (Figure S24l)^[^[^19^](#_ENREF_21)^]^, in line with the peaks assigned to N-CQDs. Therefore, it confirms the N-CQDs can modified on electrodes surface during PEC operation, which protects the electrodes from corrosion.^[^[^22^](#_ENREF_25)^]^

**Table S3**. Charge transfer resistance (*R*_ct_) and solution resistance (*R*) in the one-compartment cell with different electrolytes.

|  | ***R*/ Ω** | ***R*_ct_/ kΩ** |
| --- | --- | --- |
| **KHCO_3_** | 340.6 | 172.9 |
| **Buffer** | 434.1 | 24.1 |
| **N-CQDs** | 407.2 | 22.8 |

**Table S4**. Performance comparison of the PEC systems in this work with reported unbiased dual-photoelectrode PEC cells.

| System | Irradiation source | Electrolyte | Electrode area (cm^2^) | Current  (mA) | | H_2_O_2_ yield  (1 h) | Electricity output | Ref. |
| --- | --- | --- | --- | --- | --- | --- | --- | --- |
| Two-compartment Photoanode: BiVO_4_  photocathode: Cu_2_O | λ= 450.0 nm LED lamp (2.6 mW cm^−2^) | Photoanode electrolyte: N-CQDs, pH = 8.0; photocathode electrolyte: pH = 8.0 | 6 | 1 | 1380.0  μmol·L^–1^ | | *V*_oc_: 0.43 V, *P*_max_:  0.21 mW·cm^−2^ | This work |
| Two-compartment Photoanode: Mo-doped BiVO_4_  photocathode: p-BVO/MO_x_/NiNC | AM 1.5G  light (100.0 mW cm^−2^) | Photoanode electrolyte: 1 M NaHCO_3_, pH = 8.3;  photocathode electrolyte: 0.5 M Na_2_SO_4_, pH = 6.0 | 9 | 1 | 338.8 μmol·L^–1^ | | / | [[23](#_ENREF_26)] |
| Two-compartment Photoanode: NiFeO_x_/BiVO_4_  photocathode: polyterthiophene (pTTh) | AM 1.5G  light (100.0 mW cm^−2^) | Photoanode electrolyte: 0.1 M KOH, pH = 13.0;  photocathode electrolyte: 1 M potassium borate buffer, pH = 9.5 | 9 | 10 | 6.4  mmol·L^−1^ | | / | [[24](#_ENREF_27)] |
| Two-compartment Photoanode: Ruthenium catalyst-decorated TiO_2_ nanorods (RuO_x_/TNR)  photocathode: anthraquinone-anchored graphite rods (AQ/G) | AM 1.5G  light (100.0 mW cm^−2^) | Photoanode electrolyte: 1 M H_2_SO_4_, pH = 0;  photocathode electrolyte: 1 M KOH, pH = 14.0 | 4 | 3 | 0.8 mmol·L^−1^ | | / | [[25](#_ENREF_28)] |
| One-compartment Photoanode: TiO_2_  photocathode: polyterthiophene (pTTh) | AM 1.5G (100.0 mW cm^−2^) | 1 M NaOH,  pH = 14.0 | Photoanode: 1.8 cm^2^, photocathode: 1.6 cm^2^ | 0.3 | / | | *V*_oc_: 0.90 V, *P*_max_:  0.22 mW·cm^−2^ | [[7](#_ENREF_7)] |

Clearly, our system can generate H_2_O_2_ and electricity at much milder conditions than other systems, and its performance is also above the average of these systems. Overall, our system has several advantages: 1) Un-modified electrodes are used, simplifying the electrode fabrication; 2) Milder PEC conditions, thus avoiding highly basic or highly acidic conditions that can lead to serious corrosion of the reactor and instability of the photoelectrodes; 3) The novel use of a semiconductor electrolyte that allows selective modulation of both H_2_O_2_ yield and electricity output with the same photoelectrodes, this cannot easily be achieved using traditional electrolytes; 4) The semiconductor electrolytes can be prepared from cheap starting materials (the cost of betaine-type Meldonium is 30 yuan/g) and even if the cost is slightly higher than for traditional electrolytes and buffers, the efficiency, selectivity as well as the stability is vastly improved.

**Movie S1.** Videos of the demo for lighting a LED lamp under light irradiation (λ at 450 nm with input optical power of 2.60 mW cm^−2^)

**3. References**

[1] K.-H. Ye, Z. Wang, J. Gu, S. Xiao, Y. Yuan, Y. Zhu, Y. Zhang, W. Mai, S. Yang, *Energy Environ. Sci.* **2017**, *10*, 772-779.

[2] X. Chang, T. Wang, Z. J. Zhao, P. Yang, J. Greeley, R. Mu, G. Zhang, Z. Gong, Z. Luo, J. Chen, Y. Cui, G. A. Ozin, J. Gong, *Angew. Chem. Int. Ed.* **2018**, *57*, 15415-15419.

[3] J. Liu, D. Li, K. Zhang, M. Yang, H. Sun, B. Yang, *Small* **2018**, *14*, 1703919.

[4] Z. Bao, J. Zhao, S. Zhang, X. Peng, Y. Shao, C. Jiang, Z. Xu, X. Zhong, Z. Yao, J. Wang, *Nano Res.* **2023**, *16*, 9050-9058.

[5] R. Miao, S. Zhang, J. Liu, Y. Fang, *Chem. Mater.* **2017**, *29*, 5957-5964.

[6] F. Chen, Q. Qing, J. Xia, J. Li, N. Tao, *J. Am. Chem. Soc.* **2009**, *131*, 9908-9909.

[7] B. Zhang, L. He, T. Yao, W. Fan, X. Zhang, S. Wen, J. Shi, C. Li, *ChemSusChem* **2019**, *12*, 1026-1032.

[8] P. Yin, T. Yao, Y. Wu, L. Zheng, Y. Lin, W. Liu, H. Ju, J. Zhu, X. Hong, Z. Deng, G. Zhou, S. Wei, Y. Li, *Angew. Chem. Int. Ed.* **2016**, *55*, 10800-10805.

[9] B. Geng, J. Hu, Y. Li, S. Feng, D. Pan, L. Feng, L. Shen, *Nat. Commun.* **2022**, *13*, 5735.

[10] a) J. Jian, S. Wang, Q. Ye, F. Li, G. Su, W. Liu, C. Qu, F. Liu, C. Li, L. Jia, A. A. Novikov, V. A. Vinokurov, D. H. S. Harvey, D. Shchukin, D. Friedrich, R. van de Krol, H. Wang, *Adv. Mater.* **2022**, *34*, 2201140;b) C. Zhang, C. Xie, Y. Gao, X. Tao, C. Ding, F. Fan, H. L. Jiang, *Angew. Chem. Int. Ed.* **2022**, *61*, e202204108.

[11] K. Zhang, J. Liu, L. Wang, B. Jin, X. Yang, S. Zhang, J. H. Park, *J. Am. Chem. Soc.* **2020**, *142*, 8641-8648.

[12] F. Yuan, T. Yuan, L. Sui, Z. Wang, Z. Xi, Y. Li, X. Li, L. Fan, Z. a. Tan, A. Chen, M. Jin, S. Yang, *Nat. Commun.* **2018**, *9*, 2249.

[13] a) J. Sun, Y. Wang, S. Guo, B. Wan, L. Dong, Y. Gu, C. Song, C. Pan, Q. Zhang, L. Gu, F. Pan, J. Zhang, *Adv. Mater.* **2020**, *32*, 1906499;b) X. Zhang, Z. Shao, X. Zhang, Y. He, J. Jie, *Adv. Mater.* **2016**, *28*, 10409-10442.

[14] J. Premkumar, *Chem. Mater.* **2004**, *16*, 3980-3981.

[15] Z. Tian, Z. Wang, Z. Ma, J. Li, X. Wang, *Sci. China Mater.* **2024**, *67*, 1564-1573.

[16] T. F. Yeh, C. Y. Teng, S. J. Chen, H. Teng, *Adv. Mater.* **2014**, *26*, 3297-3303.

[17] Y. Wang, L. Li, G. Li, Q. Zhao, X. s. Wu, Y. Wang, Y. Sun, C. Hu, *ACS Catal.* **2023**, *13*, 6486-6496.

[18] B. Q. Li, C. X. Zhao, J. N. Liu, Q. Zhang, *Adv. Mater.* **2019**, *31*, 1808173.

[19] T. B. Song, Z. H. Huang, X. R. Zhang, J. W. Ni, H. M. Xiong, *Small* **2023**, *19*, 2205558.

[20] Z. Wang, P. Zhang, C. Yin, Y. Li, Z. Liao, C. Yang, H. Liu, W. Wang, C. Fan, D. Sun, L. Cheng, *Adv. Funct. Mater.* **2023**, *33*, 2300341.

[21] a) H. Ren, F. Qi, A. Labidi, J. Zhao, H. Wang, Y. Xin, J. Luo, C. Wang, *Appl. Catal. B-Environ. Energy* **2023**, *330*, 122587;b) X. Tao, M. Liao, F. Wu, Y. Jiang, J. Sun, S. Shi, *Chem. Eng. J.* **2022**, *443*, 136442.

[22] H. Zhang, S. Li, L. Xu, R. Momen, W. Deng, J. Hu, G. Zou, H. Hou, X. Ji, *Adv. Energy Mater.* **2022**, *12*, 2200665.

[23] S. Shi, Y. Song, Y. Jiao, D. Jin, Z. Li, H. Xie, L. Gao, L. Sun, J. Hou, *Nano Lett.* **2024**, *24*, 6051-6060.

[24] W. Fan, B. Zhang, X. Wang, W. Ma, D. Li, Z. Wang, M. Dupuis, J. Shi, S. Liao, C. Li, *Energy Environ. Sci.* **2020**, *13*, 238-245.

[25] T. H. Jeon, B. Kim, C. Kim, C. Xia, H. Wang, P. J. J. Alvarez, W. Choi, *Energy Environ. Sci.* **2021**, *14*, 3110-3119.
